# Recurrent acquisition of nuclease-protease pairs in antiviral immunity

**DOI:** 10.1126/science.aea8769

**Published:** 2025-11-13

**Authors:** Owen T. Tuck, Jason J. Hu, Santiago C. Lopez, Benjamin A. Adler, Claire E. O’Brien, Kendall Hsieh, Charlotte Meredith, Kenneth J. Loi, Peter H. Yoon, Erin E. Doherty, Arushi Lahiri, Jennifer A. Doudna

**Affiliations:** 1 Department of Chemistry, University of California, Berkeley, Berkeley, USA; 2 Innovative Genomics Institute, University of California, Berkeley, Berkeley, USA; 3 Department of Molecular and Cell Biology, University of California, Berkeley, Berkeley, USA; 4 California Institute for Quantitative Biosciences (QB3), University of California, Berkeley, Berkeley, USA; 5 Howard Hughes Medical Institute, University of California, Berkeley, Berkeley, USA; 6 Molecular Biophysics and Integrated Bioimaging Division, Lawrence Berkeley National Laboratory, Berkeley, USA; 7 Li Ka Shing Center for Genomic Engineering, University of California, Berkeley, Berkeley, USA; 8 Gladstone Institute of Data Science and Biotechnology, San Francisco, USA; 9 Gladstone-UCSF Institute of Genomic Immunology, San Francisco, USA

## Abstract

Antiviral immune systems diversify by integrating new genes into existing pathways, creating new mechanisms of viral resistance. We identified genes encoding a predicted nuclease paired with a trypsin-like protease repeatedly acquired by multiple, otherwise unrelated antiviral immune systems in bacteria. Cell-based and biochemical assays revealed the nuclease is a proenzyme that cleaves DNA only after activation by its partner protease. Two distinct immune systems, Hachiman and AVAST, use the same mechanism of proteolytic activation despite their independent evolutionary origins. Examination of nuclease-protease inheritance patterns identified caspase-nuclease (*canu*) genomic loci that confer antiviral defense in a pathway reminiscent of eukaryotic caspase activation. These results uncover the coordinated activities of pro-nucleases and their activating proteases within different immune systems and show how coevolution enables defense system innovation.

Continual infection by viruses drives the evolution of widespread antiviral immune systems in bacteria and archaea (1-4). Evolutionarily independent antiviral systems including CRISPR-Cas (5), CBASS (6), Avs (2), pAgo (7), retrons (8), Lamassu (4) and Hachiman (9, 10) employ a small, interchangeable pool of genes that are typically fused to core immunity genes to provide new or alternative functionalities (3, 11-15). The acquisition of such fusion proteins represents a common but underappreciated mechanism of immune diversification through modular evolution (3, 11-15). However, it remains unclear whether newly acquired genes confer similar antiviral activities in the context of distinct antiviral systems, and whether gene acquisition might enable the discovery of new defense pathways (13, 16).

We identified multiple independent examples of bacterial immune systems with co-occurring genes encoding a trypsin-like protease and a predicted nuclease belonging to the metallo-β-lactamase (MBL)-type hydrolase superfamily (17-19). In bacterial antiphage systems, MBL hydrolases resemble nucleases from DNA uptake pathways (20, 21). While the predicted nuclease is always encoded by a separate, typically upstream gene, the protease occurs as a fusion with different antiviral defense-associated enzymes. First described in AVAST (antiviral ATPase/NTPase of the STAND superfamily, Avs) (2), the nuclease-protease gene pair also occurs in Lamassu (4), Hachiman (22) and DRT (defense-associated reverse transcriptase) antiviral systems (23). How encoded nuclease and protease enzymes contribute to defense in diverse antiviral contexts is unknown.

Here we present evidence for a unified nuclease-protease mechanism common to multiple antiviral systems with different infection-sensing mechanisms in bacteria. Cell-based and biochemical assays show that the nuclease exists initially as an enzymatically inactive ‘gated’ proenzyme. Site-specific cleavage by the partner protease liberates the nuclease active site. We show that the activated nuclease is specific for DNA and executes indiscriminate DNA cleavage to restrict viral replication. This mechanism is similar in otherwise unrelated immune systems, suggesting that coordination between the nuclease and protease is conserved in immune systems with different modes of infection sensing. Phylogenetic analysis revealed that gated nuclease homologs additionally exist in genomic loci alongside caspase-family proteases. We found that these caspase-nuclease (*canu)* loci encode defense systems in which the caspase activates the downstream nuclease, suggesting parallel co-evolution of nucleases with multiple distinct partner proteases. Beyond revealing how paired nuclease and protease enzymes function in multiple antiviral systems, these results demonstrate that modular immune domains can guide the discovery and functional annotation of immune pathways, including systems like *canu* that display unexpected parallels with eukaryotic caspases.

## Results

### Repeated acquisition of paired genes in multiple antiphage systems

**Fig. 1.**
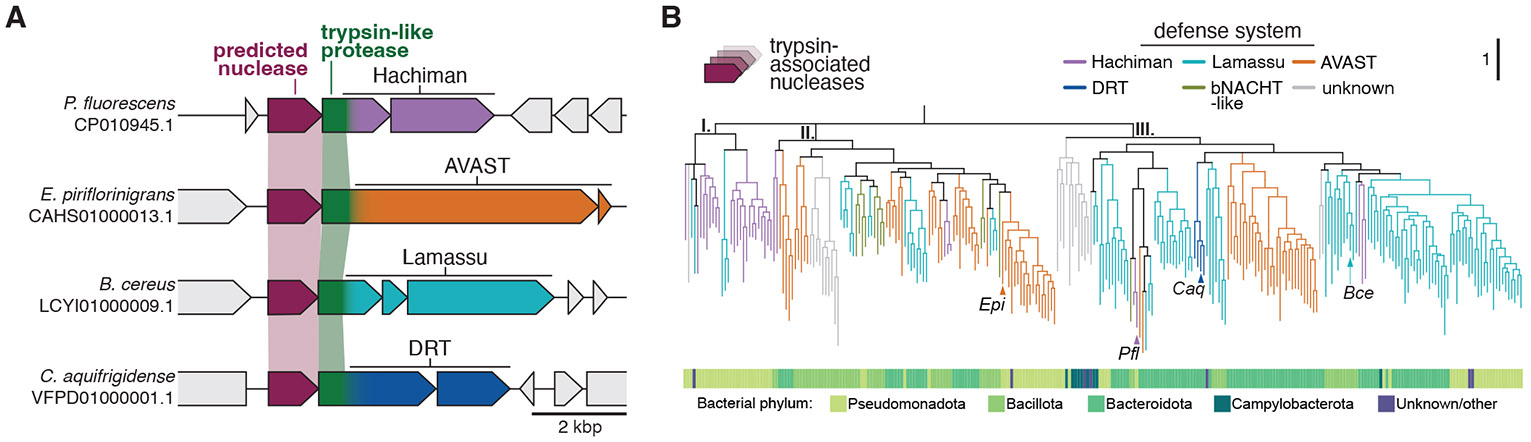
Multiple defense systems acquired a predicted nuclease and fused protease. (**A**) Loci illustrating the genetic architecture of multiple defense systems encoding a predicted N-terminal MBL hydrolase-like nuclease and a fused protease. (**B**) Phylogenetic tree of MBL hydrolase-like nucleases accessory to known defense systems which are associated with fused trypsin-like protease domain. Tree leaves are colored according to inclusion of the nuclease in known defense systems based on neighborhood analysis. The outer track indicates bacterial phyla for each nuclease representative. Leaves shown in (A) are labelled. Branches with support values < 60 are deleted. A subtree is shown. For the complete phylogeny, see fig. S2A and B.

We noticed that some Hachiman, AVAST, Lamassu and DRT antiphage defense systems share a pair of co-occurring genes. In each case, a gene encoding a predicted nuclease, belonging to the MBL-fold hydrolase superfamily, sits immediately upstream of a gene encoding a trypsin-like protease fused to the N-terminus of a defense-associated enzyme (i.e. HamA, Avs1a or LmuA) (Fig. 1A). Across all four systems, the nuclease and protease co-occur and share sequence and structural homology (fig. S1), suggesting they are evolutionarily and functionally linked.

To determine how antiphage systems acquired nuclease-protease enzyme pairs, we performed sequence- and structure-based searches for homologs of the predicted nuclease, followed by genomic context mining (fig. S2, see methods). Our analyses revealed a phylogenetic pattern consistent with frequent exaptation and horizontal genetic exchange (Fig. 1B). With the exception of rare DRT variants which emerged only once, all systems acquired the nuclease-protease pair on multiple independent occasions. Reconciliation analyses and comparison between nuclease and species trees supports transfer of these genes between defense systems (Fig. S3A, see methods). Moreover, nuclease and trypsin-like protease gene trees are similar, consistent with coevolution of nuclease-protease pairs (Fig. S4A). Our analyses reveal transfer of paired nuclease and protease genes within known defense systems (Fig. 1B), and identify additional genomic loci with potential immune functions (fig. S2C).

### Hachiman-regulated proteolytic cleavage unmasks the nuclease active site

Our analyses indicate that prokaryotic immune systems frequently acquire nuclease-protease enzyme pairs from other, unrelated systems. To investigate how such acquisition alters immune system function, we began by examining examples of nuclease-protease gene integration into the Hachiman immune system. In ~5% of Hachiman systems, the protease domain is fused to HamA, the nuclease responsible for activation-dependent DNA degradation (figs. S5A, S6A). Analysis of diverse HamA sequences revealed multiple independent examples of protease-HamA fusions, consistent with the corresponding nuclease phylogeny (Fig. 1B; figs. S6B-G, S7A). Notably, other types of HamA fusions each occurred just once during Hachiman evolution, but do not co-occur with nuclease genes and are predicted to confer diverse biochemical functions (fig. S6 and fig. S7A-C) (9, 10, 24). Furthermore, in virtually all the protease-HamA fusions, the canonical D-GEXK catalytic motif required for HamA nuclease activity is absent (fig. S7D). This evolutionary pattern implies that fusion Hachiman systems arose independently and lost HamA activity through mutation of active site residues.

**Fig. 2.**
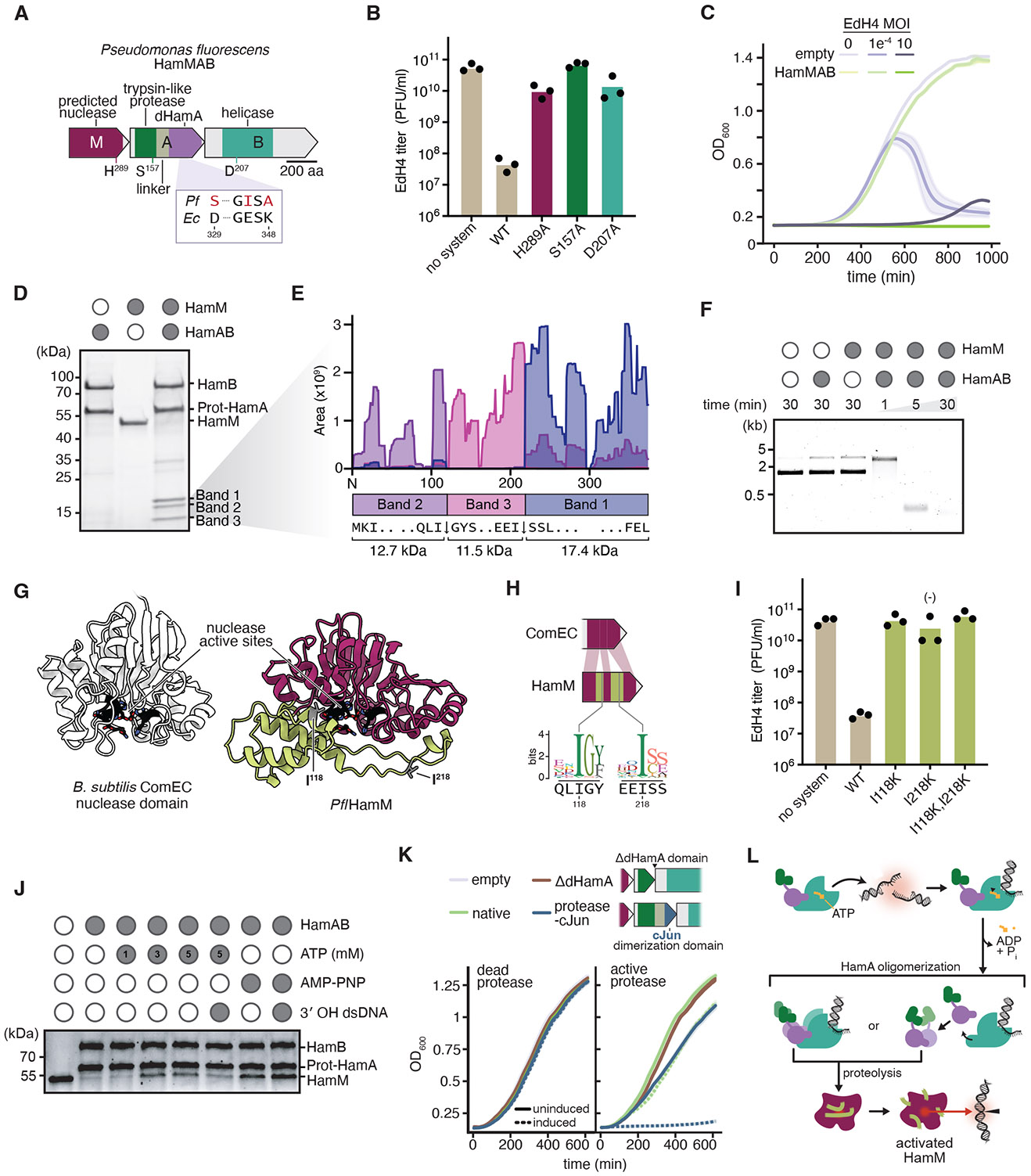
HamMAB encodes a specific protease which activates a partner nuclease. (**A**) *Pseudomonas fluorescens* HamMAB operon (CP010945.1) annotated with predicted catalytic residues and comparison to ECOR31 HamA active site. (**B**) Quantification of phage EdH4 plaque assays against *Pfl*HamMAB and mutants. Individual data points of three independent biological replicates are shown along with the mean. (**C**) Growth curves of *E. coli* expressing *Pfl*HamMAB and empty control during EdH4 infection at specified multiplicity of infection (MOI). Data are shown as mean ± standard deviation (shaded area) across three independent biological replicates. (**D**) *In vitro* reactions of HamM and HamAB visualized on a Coomassie PAGE gel. (**E**) Mass spectrometry reads of fragment 1, fragment 2, and fragment 3 mapped onto the HamM sequence. (**F**) *In vitro* plasmid clearance assay of HamM and HamAB with time course. (**G**) Comparison of AlphaFold3 models of the *Bacillus subtilis* ComEC MBL hydrolase-like nuclease domain (uniprot: P39695) with HamM, with active site colored black, protease cut sites colored gray, and insertions colored lime green. (**H**) Top: cartoon showing location of insertions in the HamM primary sequence and proteolysis sites. Bottom: sequence logos of cut site position among related nucleases, with the *Pfl*HamM primary sequence as a reference. (**I**) Quantification of phage EdH4 plaque assays against *Pfl*HamMAB and protease cut site mutants. Individual data points of three independent biological replicates are shown along with the mean. (**J**) *In vitro* reactions of HamM and HamAB with varying concentrations of ATP, the non-hydrolyzable analog AMP-PNP, and 3’ overhang dsDNA visualized on a Coomassie PAGE gel. (**K**) Growth curves of *E. coli* expressing variant HamMAB loci either lacking the HamA domain or lacking the HamA domain and fused to the c-Jun dimerization peptide. Left, growth curves in the protease inactive *Pfl*HamMAB background (S157A). Right, growth curves in the wildtype *Pfl*HamMAB background. A solid line represents no induction, while the dashed line represents induction. Data are shown as mean ± standard deviation (shaded area) across three independent biological replicates. (**L**) Model for HamMAB defense, where DNA sensing activates ATP hydrolysis, which leads to oligomerization of protease-HamA and subsequent proteolytic activation of HamM by removal of gating loops.

We cloned and expressed the *Pseudomonas fluorescens* nuclease-protease Hachiman locus (*Pfl*HamMAB) in *E. coli* and verified that it provides defense against phage EdH4 and related phages, reducing viral titer substantially (Fig. 2A, B; fig. S8). Single amino acid substitutions of predicted active site residues in the HamM predicted nuclease, the protease domain of HamA and the HamB helicase each abolished immunity, suggesting that nuclease, protease and helicase activities are all essential for antiphage activity (Fig. 2B; fig. S9). Despite toxicity upon expression, nuclease-protease Hachiman prevented cell lysis at low multiplicity of infection, while high viral doses led to cell culture collapse (Fig. 2C; fig. S10). These findings suggest that *Pfl*HamMAB, like canonical HamAB systems, defends against phage by inducing programmed cell death or growth arrest.

To determine how nuclease-protease activities enable phage defense, we purified the HamM protein and the HamAB protein complex separately and tested their interactions *in vitro* (fig. S11). Incubation of HamM with HamAB resulted in the appearance of three proteolytic fragments and the disappearance of a 42 kDa species corresponding to full length HamM (Fig. 2D). Mass spectrometry analysis mapped these fragments to specific regions of HamM, with two cleavage sites occurring immediately C-terminal to isoleucine residues I118 and I218 (Fig. 2E). Proteolysis was absent when we incubated HamAB with other proteins (fig. S12). These data support the conclusion that the trypsin protease domain of HamA functions as an endopeptidase specific for certain isoleucine residues in HamM. Functional assays further demonstrated that plasmid incubation with either HamM or HamAB alone induced plasmid DNA nicking, whereas incubation with HamM and HamAB together resulted in complete DNA degradation (Fig. 2F). HamM together with HamAB also degrades short dsDNA, ssDNA, and isolated phage EdH4 DNA, but does not cleave RNA (fig. S13). These findings support the conclusion that proteolytic cleavage of HamM by protease-HamA converts HamM into a potent deoxyribonuclease.

To understand how proteolysis activates HamM nuclease activity, we compared the AlphaFold-predicted structure of HamM to a homologous MBL hydrolase-type nuclease involved in natural competence (20, 25). Superposition suggested that HamM includes two disordered insertions relative to the canonical nuclease, both of which appear to block the nuclease active site (Fig. 2G; fig. S1). One cleavage-site isoleucine is located on each insertion; both are highly conserved at their respective positions (Fig. 2H). Hypothesizing that proteolysis removes the active site blockade created by insertions, we made single and double amino acid substitutions of cleavage site residues and tested phage defense. Mutation of either isoleucine residue is sufficient to abolish defense (Fig. 2I; fig. S9B). Our results suggest that HamM is a ‘gated’ pro-nuclease that acquired regulatory insertions responsive to a partner protease. To further confirm that cleavage of insertion regions activates the pro-nuclease, we engineered synthetic breaks into the HamM backbone at identified sites of proteolysis. The resulting ‘pre-cut’ nuclease is constitutively toxic to host cells, even when its partner protease is catalytically deactivated (fig. S14).

### DNA sensing and oligomerization trigger HamMAB defense

HamM is a pro-nuclease activated by a protease fused to HamA, but the mechanism of protease-HamA regulation remains unclear. We reasoned that DNA damage or DNA intermediates generated during phage replication triggers HamMAB immune activity, akin to canonical Hachiman systems. To test this hypothesis, we treated cells expressing HamMAB with subinhibitory concentrations of nalidixic acid, a DNA-damaging antibiotic. We observed a mild growth defect suggestive of genome integrity sensing, in line with prior work on Hachiman systems (fig. S15) (26, 27). To gain molecular insight into immune regulation and activation, we tested the effect of different ATP concentrations on HamM proteolysis. Consistent with evidence from canonical and fusion Hachiman systems (26, 28), we found that ATP inhibits immune activation: upon exposure to DNA substrates, ATP hydrolysis led to increased HamM activation (Fig. 2J; fig. S16). In support of the role for ATP hydrolysis in Hachiman activation, treatment with a non-hydrolyzable ATP analog (AMP-PNP) stalled HamM processing even when activating DNA was present (Fig. 2J).

Given that HamAB activates upon DNA sensing and ATP hydrolysis, we sought to determine the role of the dHamA domain in triggering HamM. HamA forms a complex with HamB and therefore likely controls its fused protease (10, 26, 28). We removed the dHamA domain and tested for growth defects in cells. Notably, no toxicity was observed, suggesting an alternative role for HamA during activation (Fig. 2K). Because oligomerization was shown to activate fused effectors in related systems (29-31), we examined whether dHamA induces protease oligomerization by replacing it with the leucine zipper dimerization domain from the human c-Jun protein (30, 32). This protease-cJun construct results in potent toxicity, while catalytically deactivated protease-cJun-expressing cells are healthy (Fig. 2K). These data suggest the protease alone is insufficient for nuclease activation, and that HamA-mediated oligomerization is necessary for proteolysis. In summary, we propose that upon DNA sensing, HamB hydrolyzes ATP and triggers protease-HamA, leading to HamA oligomerization and proteolytic cleavage of HamM, unmasking the nuclease active site and unleashing nuclease activity (Fig. 2L) (9, 10, 28).

### Gated nuclease activation is conserved in AVAST

**Fig. 3.**
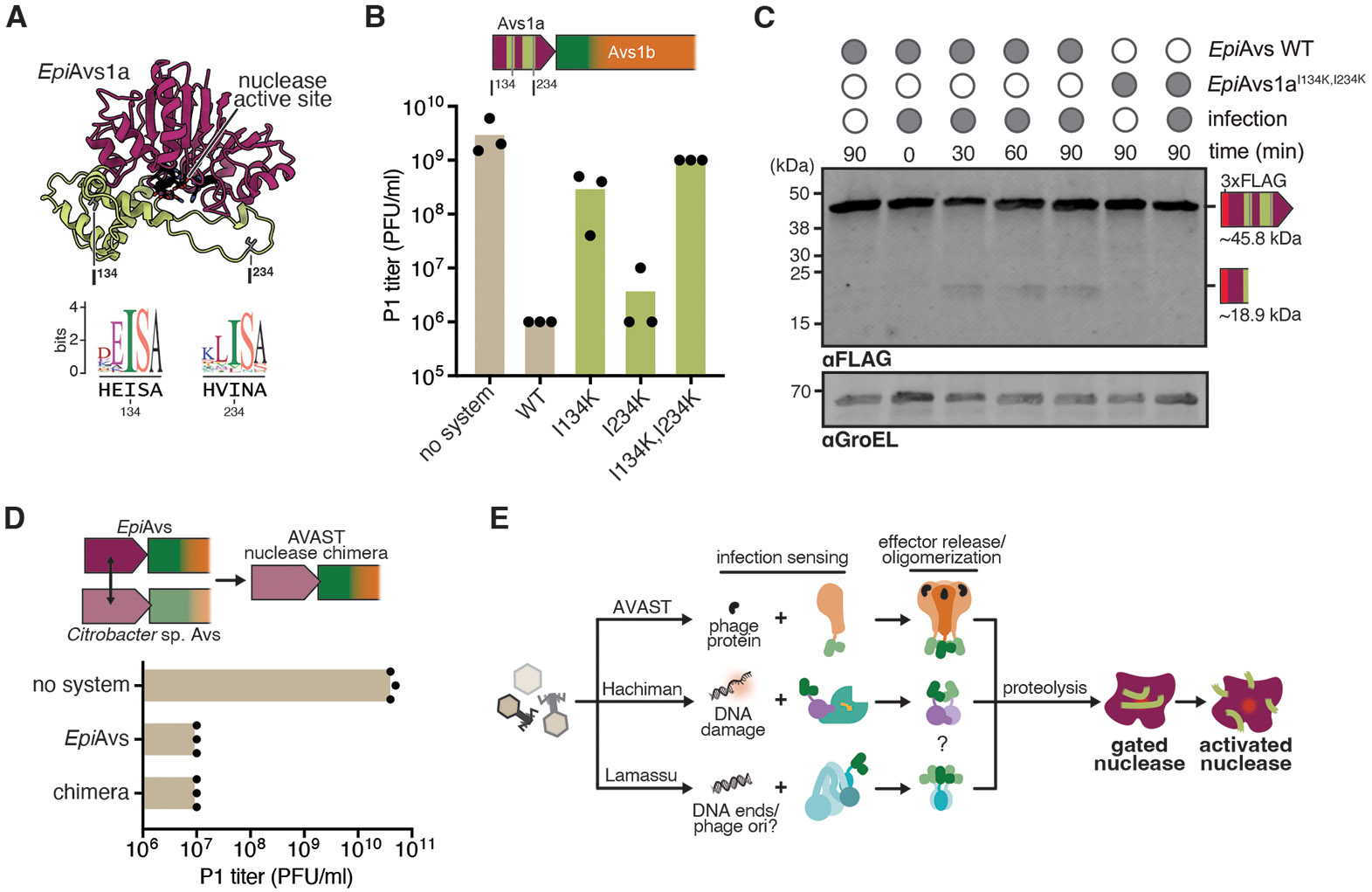
Nuclease-protease pairs function in AVAST. (**A**) AlphaFold3 model of the AVAST Avs1a nuclease from *E. piriflorinigrans* (CFBP5888), with active site colored black, predicted protease cut sites colored gray, and insertions colored lime green. Below, sequence logos of predicted cut site positions among related nucleases, with the *Epi*Avs1a primary sequence as a reference. (**B**) Quantification of phage P1 plaque assays against *Epi*Avs and predicted protease cut site mutants. Individual data points of three independent biological replications are shown along with the mean. A portion of the locus showing locations of insertions in the *Epi*Avs1a primary sequence and predicted cut sites is shown. (**C**) Immunoblot of cell lysates from cells expressing *Epi*Avs or *Epi*Avs with cut site mutants in the Avs1a nuclease (I134K, I234K) infected with phage P1 (MOI 3). Both constructs encode a 3xFLAG-tag fused to the N-terminus of Avs1a. GroEL serves as a loading control. The full length protein (~45.8 kDa) and the predicted cleavage product (18.9 kDa) are indicated. (**D**) Quantification of phage P1 plaque assays against *Epi*Avs and a chimeric *Epi*Avs system with an Avs1a nuclease from *Citrobacter* sp. ESBL3 (NZ_JAZEVZ010000002.1). Individual data points of three independent biological replications are shown along with the mean. (**E**) Model for a conserved mechanism of pro-nuclease activation among AVAST, Hachiman, and Lamassu defense systems. Upon infection sensing, defense system components oligomerize, triggering the protease and subsequent proteolytic removal of gating loops in co-encoded nucleases.

We wondered whether nuclease-protease enzyme partners retain functional properties despite their presence in divergent defense systems. To test this possibility, we investigated the nuclease-protease pair in an AVAST antiphage system from *Erwinia piriflorinigrans* (*Epi*Avs, Fig. 1), a phage-protein sensing antiphage system with otherwise no homology to Hachiman (2, 29). We confirmed potent defense against phage P1 and specific activation in response to expression of viral terminase, consistent with prior work (Fig. 3A, B; fig. S17). Structural prediction of the AVAST nuclease homolog (*Epi*Avs1a) shows dual insertions that appear to block the conserved active site, including two conserved isoleucine residues, analogous to structural features of *Pfl*HamM (Fig. 3A). We constructed single and double amino acid substitutions of conserved isoleucine residues and tested for AVAST-mediated defense against phage P1 in *E. coli* (2). Each mutation alone reduced defense, while the double mutation eliminated AVAST-mediated protection entirely (Fig. 3B; fig. S18). To confirm that nuclease is cleaved by the Avs1b protease during infection, we constructed N-terminally tagged versions of *Epi*Avs1a and monitored cleavage by western blot. A cleavage product corresponding with proteolysis adjacent to isoleucine 134 appeared upon infection, but was absent in cut site mutants (Fig. 3C). These data suggest that cleavage of a single gating loop is sufficient to activate the pro-nuclease, consistent with phage defense data (Fig. 3B, C).

Avs1b activates a ‘gated’ effector nuclease via proteolysis, despite considerable phylogenetic distance from *Pfl*HamMAB and a different infection sensing mechanism (Fig. 1B). To demonstrate transferability of nuclease-protease systems implied by phylogenetic analyses, we constructed a chimeric *Epi*Avs encoding a nuclease transplanted from a *Citrobacter* Avs system. This chimeric system displays equivalent defense against phage (Fig. 3D, fig. S18). Activation mechanism conservation in AVAST and Hachiman systems implies nuclease-protease gene pairs behave similarly in other antiphage systems (Fig. 3E). Accordingly, oligomerization upon infection sensing has been shown to activate fused effectors in Hachiman (Fig. 2K) (28), AVAST (29) and Lamassu systems (30, 31) (Fig. 3E). Disordered loops proximal to the active site are present in every antiphage trypsin-associated nuclease identified in this study (fig. S19).

### Identification of a caspase-nuclease defense system

**Fig. 4.**
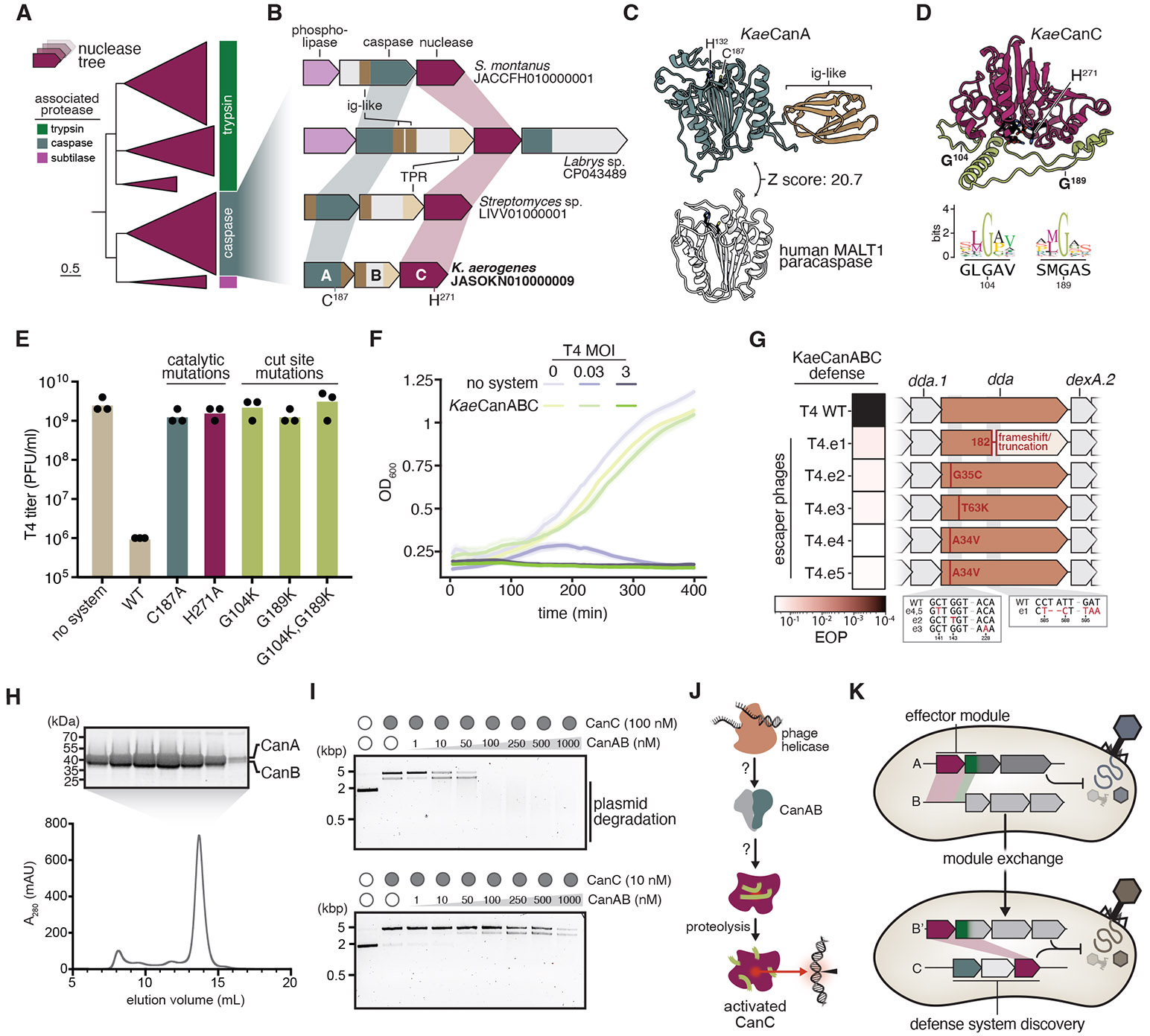
*Canu* is an antiphage defense system with a caspase-activated nuclease. (**A**) Phylogenetic tree showing major clades of nucleases associated with multiple protease families, as annotated by the outer track (right). A subtree is shown. For the complete phylogeny, see fig. S2. (**B**) Representative caspase-nuclease or *canu* loci with domains annotated (ig-like, immunoglobulin-like; TPR, tetratricopeptide repeat). The *Klebsiella aerogenes* CanABC operon (bottom) is annotated with predicted catalytic residues. (**C**) Structural homology (DALI Z-score: 20.7) between the AlphaFold3 model of *Kae*CanA and the human paracaspase MALT1 (Mucosa-associated lymphoid tissue lymphoma translocation protein 1, PDB: 3UOA), with active sites colored black. (**D**) AlphaFold3 model of *Kae*CanC, with active site colored black, predicted protease cut sites colored gray, and insertions colored lime green. Below, sequence logos of predicted cut site positions among related nucleases, with the *Kae*CanC primary sequence below. (**E**) Quantification of phage T4 plaque assays against *Kae*CanABC and mutants shown in (A). Individual data points of three independent biological replicates are shown along with the mean. (**F**) Growth curves of *E. coli* expressing *Kae*CanABC and an empty vector control during T4 infection at the specified MOI. Data are shown as mean ± standard deviation (shaded area) across three independent biological replicates. (**G**) Characterization of phage T4 mutants that escape *Kae*CanABC defense. Left, heatmap showing efficiency of plaquing (scale below) of wildtype phage T4 and five isolated escapee T4 mutants. Right, corresponding genotypes of phage T4 and escapers. Amino acid substitutions are shown on loci, and DNA-level detail is shown below. All escape mutations map to the *dda* gene. (**H**) SDS-PAGE gel of fractions from indicated peak in gel filtration analysis of *Kae*CanAB coexpression. MBP-tagged CanA coelutes with CanB. (**I**) Titration of CanAB with constant concentrations of *Kae*CanC (top: 100 nM, bottom: 10 nM) incubated with plasmid. (**J**) Proposed model of *canu* defense. The CanAB complex may either directly or indirectly sense phage helicase. Upon activation, CanA cleaves the CanC pro-nuclease, leading to activation and DNA degradation. (**K**) Model for the exchange of the nuclease-protease pair (top) and a strategy to use coevolution and transfer to discover new defense loci (bottom).

Gated nucleases co-occur with fused trypsin proteases in multiple known defense systems (Fig. 1A, B). Our phylogenetic analyses unexpectedly revealed an additional radiation of nuclease homologs that co-occur with proteases belonging to other diverse families (Fig. 4A; fig. S2). The largest clade in this group includes a nuclease-encoding gene positioned downstream of a gene encoding a protease most closely related to caspases, which are essential to human innate immunity and apoptosis (18) (Fig. 4A, B). These loci are variable in organization, encoding several genes of unknown function and multiple domains associated with oligomerization and protein-protein interactions, including tetratricopeptide repeats (TPR) and immunoglobulin-like (Ig-like) folds (Fig. 4B) (33). Caspase-associated nucleases appear in immune hotspots known as ‘defense islands’ and near transposases, both signatures of immune function (fig. S2C) (34). We wondered whether these caspase-nuclease (*canu*) loci encode a similar mechanism, wherein the caspase activates its linked nuclease to provide antiphage defense.

In the prototypical three-gene *canu* system from *Klebsiella aerogenes* (*Kae*CanABC), the *canA* gene encodes a caspase domain structurally similar to the human MALT1 paracaspase, retaining the C-terminal Ig-like fold crucial for immune activation (Fig. 4B, C) (35, 36). The CanC nuclease active site is likely blocked by dual disordered insertions based on structure prediction and comparison to related enzymes (Fig. 4D; fig S18). Both gating insertions contain conserved glycine residues (Fig. 4C), leading us to hypothesize that these positions are recognized by the caspase to trigger nuclease activity.

To test Canu activity, we challenged *E. coli* heterologously expressing *Kae*CanABC. We found that *Kae*CanABC provides strong protection against phage T4 (Fig. 4E; fig. S20). Single amino acid substitutions of predicted active site residues in the caspase or nuclease domains abolished defense, suggesting that both catalytic activities are required. Mutation of either predicted proteolysis site in the nuclease was also sufficient to ablate defense. The *Kae*CanABC system overcame infection at low viral doses, but succumbed to phage when viral titer surpassed that of the host (Fig. 4F). To identify potential viral triggers of Canu, we isolated mutant phages that escape *Kae*CanABC defense (Fig. 4G; fig. S21). Sequencing of escaper phages revealed diverse missense and frameshift mutations mapping to the *dda* gene, which encodes a nonessential SF1 helicase involved in phage DNA replication, recombination, and repair (37). Together, these data imply that *Kae*CanABC carries a conserved pro-nuclease which, upon infection sensing involving phage helicase and proteolytic processing by its partner caspase, protects cells from infection by programmed cell death or growth arrest. Whether *Kae*CanABC recognizes phage helicase by direct recognition (29), or indirectly via their activities remains to be seen (26, 38).

CanA is a caspase family protease and CanC is a putative pro-nuclease. To investigate Canu activities and determine a role for CanB, we purified each protein individually. Only CanC was obtained in isolation. Coexpression of the CanA and CanB produced a soluble complex, suggesting CanB plays a potential role in modulating CanA protease activity (Fig. 4H). Upon incubating CanC with increasing concentrations of CanAB, we observed conversion of CanC into a potent nuclease (Fig. 4I). Combined, these data demonstrate the caspase CanAB complex plays a role in activating the CanC nuclease for phage defense (Fig. 4J).

## Discussion

In this study, we discovered that multiple different prokaryotic antiviral pathways employ related nuclease-protease enzyme pairs to diversify immune function. Cell-based and biochemical data showed that protease-catalyzed cleavage of a proenzyme form of the nuclease converts it to an active DNase. Once activated, the nuclease destroys cellular DNA, likely killing or arresting infected cells to halt viral replication. Unlike most cell death effectors that act individually (2, 6, 39-41), nuclease and protease genes function in tandem and co-evolve. This enzymatic partnership provides regulation that may safeguard against autoimmunity. For example, independent control over nuclease expression enables fine-tuning of its effect on cells, while fusion of partner proteases to core defense genes which require oligomerization to activate may prevent nonspecific activation (6, 30, 31, 42). Our results reveal that proteolytic activation, long recognized as central to eukaryotic immunity (43, 44), also shapes prokaryotic defense systems more extensively than previously appreciated (45-47).

Our phylogenetic analyses suggest that ancestral nucleases acquired autoinhibitory insertions containing endopeptidase sites, creating a requirement for protease partners to restore activity. The resulting protease-nuclease pairs were then transferred between otherwise unrelated antiviral systems. Despite frequent mobilization, these enzyme pairs retain the coupled mechanism of nuclease activation, reflecting their common ancestry. Distinct genetic arrangements, including relative position and standalone versus fused configurations in trypsin and caspase systems, respectively, likely trace back to this fundamental functional association. Based on these observations, it is possible that other proteolytically activated enzyme classes participate in antiviral immunity, providing similar regulatory safeguards. Hallmarks of functional coupling, including protease coevolution and active site-blocking insertions, can guide the discovery of additional enzymatic partnerships (Fig. 4K).

One example of such a discovery is the identification of caspase-nuclease (*canu*) antiphage loci, which extend the diversity of known caspase-containing immune systems in prokaryotes (33, 48-50). In humans, multiple caspase paralogs orchestrate programmed cell death by either proteolytically activating other factors (initiator caspases), or by directly targeting cellular proteins (inflammatory and executioner caspases) (43, 51, 52). Prokaryotes encode other caspase homologs with known functions in antiphage defense (48, 50), including Type III CRISPR (45-47), gasdermin/CARD (53, 54), NLR (55) and Thoeris systems (56). *Canu* systems differ by encoding Ig-like domains that also occur in human caspases (33, 35). The *canu* system from *K. aerogenes* (*Kae*CanABC) provides potent phage defense and shows striking conservation of the nuclease activation mechanism found in Hachiman and AVAST. These results suggest that the Canu caspase functions as a specific endopeptidase, analogous to eukaryotic initiator caspases (57). While *canu* diversity and functions await further exploration, conservation of nuclease activation provides a basis for determining the mechanism of *canu* defense.

## Supplementary Material

supplementary_tables_1-6

supplementary_materials

Materials and Methods

Figs. S1 to S21

Tables S1 to S6

References (58-77)
