# Supplementary material for: Recurrent acquisition of nuclease-protease pairs in antiviral immunity
Source: Science. Author manuscript; Available in PMC 2026 Jan 13. (PMC12799240; doi:10.1126/science.aea8769)
Supplement: supplementary_materials [file NIHMS2127989-supplement-supplementary_materials.docx]

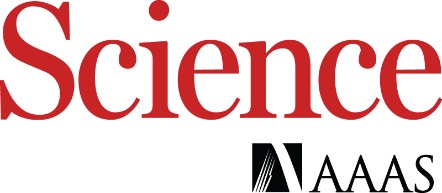


Supplementary Materials for

**Recurrent acquisition of nuclease-protease pairs in antiviral immunity**

Owen T. Tuck, Jason J. Hu, Santiago C. Lopez, Benjamin A. Adler, Claire E. O’Brien, Kendall Hsieh, Charlotte Meredith, Kenneth J. Loi, Peter H. Yoon, Erin E. Doherty, Arushi Lahiri, Jennifer A. Doudna

Corresponding author: Jennifer Doudna, doudna@berkeley.edu

**The PDF file includes:**

Materials and Methods

Figs. S1 to S21

References

**Other Supplementary Materials for this manuscript include the following:**

Tables S1 to S6

**Supplementary Materials**

**Materials and Methods**

Nuclease phylogenetic and coevolutionary analyses

Phylogenetic analyses of MBL hydrolase-like nucleases were initiated by generating a seed alignment of HamM, Avs1a and LmuA genes using ClustalOmega (*58*). The resulting alignment was manually trimmed and then used to generate a custom HMM with hmmbuild (*59*), and subsequently hmmsearch was performed on the uniprot database (2025_01). Unique HMM hits with an e-value less than 1E-3 were selected, and clustered with MMseqs2 easy-linclust using --min-seq-id 0.9 (*60*). Multiple sequence alignment using ClustalOmega was performed, followed by manual trimming. Finally, IQ-TREE was performed with the following parameters: -bb 1000 -nm 1500 -safe, with ModelFinder used to select VT+F+R10 (*61*, *62*). The phylogenetic tree was visualized with iTOL (*63*), with branches with bootstrap values below 60 deleted. A large clade containing the ComEC hydrolase/nuclease domain was chosen as a root. A subtree of only trypsin-like protease clades is shown in Fig. 1A, and a cartoon subtree of all protease-associated domains is shown in Fig 4A.

Context analysis was performed by extracting 10 kbp up and downstream of hmmsearch hits using the NCBI Entrez tool (*64*). PADLOC was then used to annotate defense systems within the gene neighborhood (*22*). If hit proteins were encoded within a known defense system as determined by PADLOC, these were annotated as such in phylogenetic trees using iTOL annotation tools (*63*). Leaves without defense system annotations were manually inspected and labelled. Nuclease structures from *Pfl*HamM, *Epi*Avs1a, *Bce*LmuA-H, and UG9 DRT systems were predicted using the AlphaFold3 web server (*25*). Structures were aligned and visualized with matchmaker in ChimeraX (*65*).

Nuclease-protease coevolutionary analysis was performed using a filtered set of nuclease-proteases with genomic contexts of at least 20 kbp (n=191). Protein coding genes found within the neighborhood were pooled, and clustered using MMseqs easy-cluster on default parameters. To further group clusters, HHblits was used on default parameters (*66*). Clusters were merged when both query-and-target coverage were above 50, and probability was above 90. Most trypsin proteases (n=164) fell under a single group. Remaining proteases that fell outside the cluster (n=36) were retrieved by an hmmsearch using an alignment of the clustered proteases generated using MAFFT- E-INS-I (*67*). One nuclease containing locus did not have a clear trypsin protease homolog, and was excluded from further analysis. Alignments of nucleases and proteases were made using MAFFT-E-INS-I, and nuclease-protease pairs were removed from further analysis if either genes appeared to be incomplete or inactive. This resulted in a set of 181 nuclease-protease pairs used as input for nuclease and protease trees. To generate a species tree, the latest taxonomic metadata and phylogenetic tree (bac120.tree) were retrieved from the GTDB database. The bac120.tree was further pruned to only display leaves associated with relevant genomes containing nuclease-protease pairs (n=160; 21 pairs had no match on GTDB).

To qualitatively assess coevolution, the phylogenetic topology of the nuclease and protease were compared to each other and to the species tree. First, the nuclease and protease alignments were trimmed and passed to IQ-TREE to generate trees for each of the respective genes using: -m MFP -bb 1000 -alrt 1000. Next, the resulting gene trees and the pruned species tree were passed to the program CoPhylo in pairs for comparison (*68*). This was done for the following pairs: nuclease vs. protease, nuclease vs. species, and protease vs. species. We then generated tanglegram visualizations in iTOL. Further reconciliation analysis was conducted for the gene trees versus the species tree. The nuclease and protease alignments were concatenated into a single alignment file. This concatenated alignment file and the species tree were passed to the program GeneRax to compute reconciliation likelihood, phylogenetic likelihood, and joint likelihood. The following parameters were selected: -r UndatedDTL and –geneSearchStrategy SPR and LG+G substitution model (*69*).

Hachiman HamA phylogenetic analysis

For analysis of Hachiman diversity, unique proteins from InterPro protein family IPR014976 and annotated as Hachiman antiphage defense system protein HamA in Refseq and Genbank were pooled and chosen for phylogenetic analysis. MMseqs2 easy-linclust was run on pooled sequences with --min-seq-id 0.552 (*60*). Then, an HMM was built based on the clustered sequences with hmmbuild, and subsequently hmmsearch was performed on the genbank nr nucleotide database (v274) (*59*). Unique HMM hits with an e-value less than 1E-5 were selected, and clustered with MMseqs2 easy-linclust using --min-seq-id 0.9. Multiple sequence alignment using muscle5.1 -super5 was performed on the clustered sequences, with an additional 10 homologs of the P. aquatile type IIS restriction endonuclease domain chosen as the outgroup (*9*, *70*). Truncated Hachiman variants (HamAs that are <150 amino acids) were manually removed and HamA-HamB fusions (likely due to stop codon misannotation) were removed from the sequence alignment, then re-aligned with muscle5.1 -super5. Poorly aligned regions were trimmed by trimAl with -gt 0.26 (*71*). Finally, IQ-TREE was performed with --bb 1000 -m LG+F+G4 (50). The phylogenetic tree was visualized with iTOL (*63*), with branches with bootstrap values below 60 deleted and branches annotated for taxonomy and the presence or absence of a fused domain (which were trimmed from the alignment to observe only HamA domain dynamics).

HamA were plotted by length, then uncommonly long HamAs were extracted and aligned. Representatives of each long HamA sequence were folded using Colabfold (*72*), then fused domains were manually extracted in ChimeraX (*65*). Each of the five extracted N-terminal domains were used to search for structural homologs via the Dali search server (*73*). Superimpositions were generated using the matchmaker function in ChimeraX (*65*). Sequence logos and locus visualizations were generated using Geneious Prime. Phylogenetic analyses of occurrences of HB and YTH domains outside of Hachiman systems were executed in the same manner as HamM phylogenetic analysis described above.

Bacterial strains and phages

For bacterial cultivation, *E. coli* strains maintained as 25% (v/v) glycerol stocks and stored at -80ºC were generally grown in LB Lennox media at 37ºC and 250 rpm aeration. Media was supplemented with carbenicillin (Sigma, 100 µg mL-1) or chloramphenicol (Sigma, 20 μg mL-1) where applicable. *E. coli* Top10 (invitrogen) was used as a cloning strain. For phage experiments using the *Pfl*HamMAB system, *E. coli* MDS42 was used. For *Epi*Avs and *Kae*CanABC phage experiments, we used *E. coli* K-12 MG1655. To purify proteins, *E. coli* BL21-AI (Invitrogen) was used.

Phage P1 was obtained from the American Type Culture Collection (ATCC 25404-B1). Phages from the BASEL collection were kindly provided by Dr. Alex Harms (*74*). All phages were verified through either whole-genome sequencing or targeted genotyping to check for stock cross-contamination. Wildtype phages were propagated at 37ºC in LB Lennox media using an initial MOI of approximately 0.1 and host *E. coli* BW25113. After culture collapse, a drop of chloroform was added and cultures were centrifuged at 4000 g for 15 min. The supernatant was passed through a 0.2 µm filter and stored at 4ºC. All phage titers were determined on assay hosts with an empty vector control in the p15a backbone using plaque assays (described below).

Plasmid construction

All plasmids used here were constructed using Gibson Cloning, Golden Gate Assembly or mutagenic PCR/ ligation, using PCR products purified by gel extraction (Zymo D4001). *Pseudomonas fluorescens*, *Klebsiella aerogenes,* and *Citrobacter* sp. ESBL3 loci were synthesized by Twist. For phage assays, all defense loci except AVAST were cloned under control of the pTet promoter in a P15A backbone encoding for chloramphenicol resistance. In addition to the pTet promoter, the *Kae*CanABC is also under the control of its native promoter, and experiments with this system were performed with no additional induction of protein expression. The AVAST system, cloned from the source plasmid (*2*), is under control of the bla promoter in a p15a backbone, which encodes chloramphenicol resistance. For phage terminase coexpression, the pJEX vector conferring kanamycin resistance was used for inducible control of genes upon addition of crystal violet. For protein purification purposes, loci were cloned under a T7 lactose-controlled promoter in a high copy vector encoding carbenicillin resistance. To assess the toxicity of HamM activated by dual-loop proteolysis (henceforth ‘pre-cut HamM’), we pre-programmed cuts in HamM by inserting stop codons immediately after the putative cut-site loop isoleucine residues. Concurrently, we added strong ribosome binding sites (BBa_B0034) and a start codon before resumption of the coding sequence (*75*). The resulting tripeptide is predicted by AlphaFold3 to retain the nuclease tertiary structure. For transplantation experiments, a nuclease from a *Citrobacter* sp. ESBL3 (WP_332628315.1) AVAST and a LmuA-H nuclease homolog in *Bacillus* sp. B1-b2 (WEHU01000028.1) were incorporated into the *Epi*Avs locus by Gibson Assembly.

All plasmids were sequence verified using Primordium. For a complete list of cloned sequences used in this study, see Table S1. A summary of plasmids used in this study and related sequences can be found in Table S2.

Plaque assays

Plaque assays were performed as previously described, using the double agar overlay method (*9*). In brief, 100 µL (AVAST, *Kae*CanABC) or 200 µL (*Pfl*HamMAB) of saturated overnight cultures carrying either an empty vector or the defense system of interest were added to molten LB Lennox agar (0.7% w/v agar, 60°C – henceforth, “top agar”), then the mixture was supplemented with antibiotics. For plaque assays assaying the antiviral activity of *Pfl*HamMAB against the diverse panel of phages, top agar was supplemented with anhydrotetracycline (aTc, Sigma) at a final concentration of 0.5 nM to induce expression of HamMAB. For plaque assays assaying the antiviral activity of *Pfl*HamMAB mutants against EdH4, top agar was supplemented with anhydrotetracycline (aTc, Sigma) at a final concentration of 1 nM to induce expression of HamMAB. For plaque assays assaying the antiviral activity of the AVAST and *Kae*CanABC systems, no additional inducers were added to the top agar. This mixture was poured over 1.5% w/v LB Lennox agar plates (presupplemented with antibiotic) and was allowed to cool and solidify. Phages were diluted tenfold in SM buffer (Teknova), then 2 or 2.5 µl of eight dilutions were spotted onto the top agar layer. For phage P1, a stock approximately tenfold higher in titer than that used for Fig. 3B is shown in Fig. 3D. After drying under sterile conditions, plates were incubated at 30ºC for 12-16 hours. Plates were then scanned and plaque forming units (PFU) were enumerated. Plaque assays were performed in biological triplicate, and data were analyzed using Graphpad Prism and Python.

Liquid infection assays

To assay bacterial growth in the presence or absence of phage, OD600 was monitored in a 96-well plate format in a plate reader (Biotek Cytation 5). To initiate growth, saturated overnight cultures carrying either an empty vector or the defense system of interest were seeded in a microplate (Corning 3903) at a CFU of ∼8e6 CFU per well in 200 μL of LB media supplemented with antibiotic and, when assaying the antiviral activity of *Pfl*HamMAB, with aTc at a final concentration of 0.5 nM or 0.125 nM. Phage were added to achieve the indicated MOI after separate dilution in SM buffer. Growth was monitored by measurement of OD600 every 5 minutes while shaking at 800 RPM (double orbital) at 30ºC for the indicated time span. Liquid growth assays were performed in biological triplicate, and data were analyzed using Python.

Protein expression and purification

For complex purification vectors in the native locus format, only HamA and CanA were tagged with 10xHis-MBP-TEV. After transformation into BL21-AI *E. coli*, single colonies were inoculated in TB media and were grown to an optical density of ∼0.6 then induced overnight at 16°C with 0.5 mM isopropyl-β-D-thiogalactopyranoside (IPTG) and 0.1% L-arabinose. Cells were harvested and resuspended in lysis buffer (20 mM 4-(2-hydroxyethyl)-1-piperazineethanesulfonic acid (HEPES) pH 7.7, 500 mM NaCl, 20 mM imidazole, 0.1% Triton X-100, 1 mM Tris (2-carboxyethyl)phosphine (TCEP), Complete EDTA (ethylenediaminetetraacetic acid)-free protease inhibitor (Roche), 0.5 mM phenylmethylsulfonyl fluoride (PMSF) and 10% glycerol). Cells were lysed by sonication, then clarified by centrifugation. The clarified lysate was incubated with Ni-NTA (Qiagen) resin for 1 hr. The resin was washed with wash buffer (20 mM HEPES, pH 8, KCl mM NaCl, 20 mM imidazole, 1 mM TCEP, and 5% glycerol), then bound protein was eluted with wash buffer supplemented with 300mM imidazole. Eluate was then run over an MBPTrap column (GE Healthcare), washed with MBP/SEC wash buffer (20 mM HEPES, pH 8, 150 mM KCl, 1 mM TCEP, and 5% glycerol), and eluted with MBP/SEC buffer supplemented with 10 mM maltose. Eluted protein from the MBPTrap column was treated with TEV protease overnight. Then, TEV protease-treated samples were run through Ni-NTA resin, and flow-through was collected. Flow-through was concentrated and run on a Superdex 200 10/300 GL column (Cytiva). Aliquots were concentrated and snap-frozen in liquid nitrogen for later use.

Protease-HamAB proteolytic cleavage assays

Protease cleavage assays of HamM by protease-HamAB were performed in isothermal amplification buffer (20 mM Tris-HCl, pH 8.8, 10 mM (NH)2SO4, 50 mM KCl, 2 mM MgSO4, 0.1% Tween 20). 1.4 µM of protease-HamAB was incubated with 1 µM of HamM for 90 minutes at 37 ºC. Controls were performed with protease-HamAB only and HamM only as well. Reactions were terminated with addition of 4x Laemmli buffer. Samples were boiled for 3 minutes at 95ºC, loaded on a 4-20% SDS PAGE gel, stained by Coomassie InstantBlue, and imaged on a ChemiDoc MP (BioRad). To assay effects of ATP and activating DNA on proteolytic cleavage of HamM, the assay above was modified as follows. 0.5 µM of protease-HamAB was mixed with ATP at the indicated concentration or 5mM AMP-PNP (Sigma, Adenylyl-imidodiphosphate). Activating dsDNA with 3′ overhang was added to a final concentration of 2µM where indicated. Reactions were allowed to incubate for 10 minutes at 30ºC before introduction of HamM. After addition of HamM, the reaction was allowed to proceed for 30 min, after which it was quenched and analyzed as described above.

Protease cleavage assays of SSB (Fisher Scientific), RecBCD (New England Biolabs), and Topoisomerase I (New England Biolabs) by protease-HamAB were performed in isothermal amplification buffer. All reactions were performed with a final concentration of 0.25 μg/µL SSB, 0.5 units/µL RecBCD, and 0.25 units/µL Topoisomerase I. For the indicated reactions (Fig. S8), SSB, RecBCD, and Topoisomerase I were boiled at 95˚C for 3 minutes before addition of protease-HamAB. Reactions were started by the addition of protease-HamAB to a final concentration of 0.5 µM and incubated at 30˚C for 30 minutes. Controls with SSB, RecBCD, and Topoisomerase I only were conducted as well. Reactions were stopped by addition of 4x Laemmli buffer. Samples were boiled for 3 minutes at 95˚C, loaded on a 4-20% SDS PAGE gel, stained by Coomassie InstantBlue, and imaged on a ChemiDoc MP (BioRad).

In-gel mass spectrometry

Experiments for mass spectrometry were performed in MBP/SEC buffer supplemented with 1mM MgCl2. 9.6 μM of protease-HamAB was incubated with 19.3 μM of HamM for 60 minutes at 30˚C. Reactions were terminated with addition of 4x Laemmli buffer. Samples were heated for 3 minutes, loaded on a 4-20% SDS PAGE gel, and visualized by Coomassie InstantBlue. Protein bands corresponding to proteolytic products of HamM were excised with a razor into 1 mm2 cubes. Gel pieces were washed twice with 50% (v/v) acetonitrile and 50mM ammonium bicarbonate (pH 8) for 15 minutes with shaking. Gel pieces were then dehydrated with 100% acetonitrile for 5 minutes with shaking. Then the solvent was removed, and gel pieces were allowed to air dry for 20 min. To the dry gel pieces, 10 mM TCEP and 40mM chloroacetic acid were added and incubated at 70ºC for 5 min. The gel pieces were washed again with 50% acetonitrile and 50% 50mM ammonium bicarbonate for 15 min with shaking. The gel pieces were then rehydrated in 50 mM ammonium bicarbonate and 1μg Chymotrypsin/ GluC (1:50) was added and incubated for 1 hour at room temperature, then 50mM HEPES pH 8 solution was added to cover the pieces. The samples were allowed to incubate overnight at 37ºC. Peptides were extracted from gel pieces with 25% acetonitrile and 50mM ammonium bicarbonate pH 8, then again with 100% acetonitrile for 5 min with shaking. Samples were filtered through a 0.22 µm PVDFspin column (Millipore) Peptides were dried in a speedvac apparatus to 30-60 µl total volume, then samples were acidified with 2 µl formic acid (neat).

In-gel Chymotrypsin/ GluC digested peptides were analyzed by online capillary nano LC-MS/MS using a 25 cm reverse phase column and a 10 cm precolumn fabricated in-house (75 µm inner diameter, packed with ReproSil-Gold C18-1.9 μm resin (Dr. Maisch GmbH)) that was equipped with a laser-pulled nanoelectrospray emitter tip. The precolumn used 3.0 μm packing (Dr. Maisch GmbH)). Peptides were eluted at a flow rate of 300 nL/min using a linear gradient of 2–40% buffer B in 140 min (buffer A: 0.05% formic acid and 5% acetonitrile in water; buffer B: 0.05% formic acid and 95% acetonitrile in water) in an Thermo Fisher Easy-nLC1200 nanoLC system. Peptides were ionized using a FLEX ion source (Thermo Fisher) using electrospray ionization into an Fusion Lumos Tribrid Orbitrap Mass Spectrometer (Thermo Fisher Scientific). Instrument method parameters were as follows: MS1 resolution, 120,000 at 200 m/z; scan range, 350−1600 m/z, orbitrap mode acquisition. The top 20 most-abundant ions were subjected to collision-induced dissociation with a normalized collision energy of 35%, activation q 0.25, and precursor isolation width 2 m/z. Dynamic exclusion was enabled with a repeat count of 1, a repeat duration of 30 seconds, and an exclusion duration of 20 seconds. RAW files were analyzed using PEAKS (Bioinformatics Solution Inc) with the following parameters: semi-specific cleavage specificity at the C-terminal site of R and K, allowing for 5 missed cleavages, precursor mass tolerance of 15 ppm, and fragment ion mass tolerance of 0.5 Daltons. Methionine oxidation was set as variable modifications and cysteine carbamidomethylation was set as a fixed modification. Peptide hits were filtered using a 5% FDR. Proteins with at least 2 unique peptides were filtered with a 5% FDR. Label free quantitation (LFQ) was performed using the PEAKS quantitation module and default parameters with the following exceptions: Top 2 peptides for each protein with a min of 10e4 abundance were used and the TIC was used for all normalization including technical replicates. Peptide mass spectrometry data were visualized using PrIntMap-R (*76*). See Tables S3-5 for data used to generate Fig. 2E.

Plasmid and phage gDNA clearance assays

Plasmid and phage DNA interference assays were conducted in isothermal amplification buffer. The plasmid substrate is a generic pUC19 vector (see Table S2), and the phage DNA substrate is EdH4 genomic DNA isolated as described above. DNA was diluted to 4 ng/μl. Reactions were started with addition of Protease-HamAB at 5nM and HamM at 10nM, and incubated at 30°C for 1 min, 5 min, or 30 min. Reactions were quenched with addition of proteinaseK (New England Biolabs) and 10x Bluejuice. Reactions were imaged on 1% TAE agarose gels. Gels were stained with SYBR Safe and imaged on a ChemiDoc MP (BioRad).

For *Kae*CanABC plasmid cleavage experiments, CanC was diluted to the indicated concentrations in isothermal amplification buffer with plasmid DNA diluted to 4 ng/μl. CanAB was added at the indicated concentrations, and reactions were allowed to proceed at 37ºC for 30 min before quenching by addition of proteinaseK (New England Biolabs) and 10x Bluejuice. Reactions were imaged on 1% TAE agarose gels. Gels were stained with SYBR Safe and imaged on a ChemiDoc MP (BioRad).

Protease-HamAB nuclease short nucleic acid activity assay

Short nucleic acid (ds/ssDNA, ds/ssRNA) cleavage assays were performed in isothermal amplification buffer or MBP/SEC buffer supplemented with 1mM ZnCl_2_. 0.5 µM of protease-HamAB and 0.5 µM of HamM were incubated with 0.5 µM nucleic acid for 30 minutes at 30˚C. Reactions were stopped by addition of ProteinaseK (New England Biolabs) and 10x Bluejuice. Reactions were run on a 2% agarose gel stained with SYBR Gold (Thermo Fisher) and imaged on a ChemiDoc MP (BioRad). For a summary of oligonucleotide substrates, see Table S6.

Malachite green ATPase assays

Orthophosphate liberation was determined with a Malachite Green Phosphate Assay kit (BioAssay Systems) according to the manufacturer protocol. All reactions were conducted in isothermal amplification buffer. Substrates tested included two different ssDNAs (ssDNA 1 and ssDNA 2), blunt-ended dsDNA, dsDNA with 3′ overhang, dsDNA with 5′ overhang, and plasmid DNA. DNA substrates were diluted to 100 nM, or 4 ng/µl for plasmid reactions, in a total reaction volume of 40 µL in clear bottom, flat, black 96-well assay plates (Corning Costar). Reactions were started with addition of *Pfl*HamAB to 40 nM and ATP to 1 mM, followed by incubation at 30˚C for 30 minutes. Additional controls with buffer only, ATP only, HamAB only, and no DNA substrates were performed. Reactions were quenched with the addition of activated malachite green reagent. The absorbance values of wells were measured after 20 min of color development at ambient temperature with a Biotek plate reader at 620 nm. Orthophosphate liberation was interpolated against a standard curve with known concentrations of free phosphate. For a summary of oligonucleotide substrates, see Table S6.

Nuclease and caspase structural analyses

Nuclease structures from *Pfl*HamM, pre-cut *Pfl*HamM, *Epi*Avs1a and *Kae*CanuC were predicted using the AlphaFold3 web server (*25*). Predicted structures were compared to the AlphaFold Database (AFDB) using Foldseek (*77*). A confident hit, (uniprot: P39695) protein 3 from the *B. subtilis* ComE operon, was aligned and visualized with matchmaker in ChimeraX (*65*). Sequence logos of insertion regions containing the putative protease cleavage sites as determined by mass spectrometry (see above) were generated by performing BLAST searches, then aligning 100 hit sequences with ClustalOmega (*58*) and visualizing regions of interest with the built-in sequence logo function of Geneious Prime. To generate a structural comparison of *Kae*CanuA, Dali was used to search the protein data bank for remote homology to experimentally determine structures, then superpositions were regenerated and visualized in ChimeraX using matchmaker.

Liquid toxicity assays

Liquid culture toxicity assays were performed in 96-well plate format in a plate reader (Biotek Cytation 5), and toxicity was assessed by monitoring OD600 over time. Saturated overnight cultures of the indicated strains were seeded into a microplate (Corning 3903) at a CFU of ∼8e6 CFU per well in 200 μL of LB media supplemented with antibiotics and the indicated concentration of inducer. For drug activation experiments, nalidixic acid (Sigma) or gentamicin (Sigma) were also added at the indicated sub-inhibitory concentrations as determined previously (*26*). Growth was monitored by measurement of OD600 every 5 minutes while shaking at 800 rpm (double orbital) at 30ºC for the indicated time span. Liquid growth assays were performed in biological triplicate, and data were analyzed using Python.

Western blot analyses

To assess nuclease cleavage in cells, overnight cultures of either *Epi*Avs or the cut site mutant, each with an N-terminal 3xFLAG tag, were diluted 100-fold, then incubated at 30ºC with shaking at 250 rpm until cultures reached an optical density of 0.5. Phage was then added at an MOI of 3. 300 µl aliquots were taken at the indicated time points and were lysed with the addition of 1x Laemmli buffer with 50 mM TCEP. Samples were boiled for 3 minutes at 95ºC and were then loaded on a 4-20% SDS PAGE gel. Separated proteins were transferred to a PVDF membrane (Bio-Rad, #1620177) for 1 hour at 100 V in Towbin buffer (25mM Tris base, 192mM glycine, 20% methanol). Membranes were blocked (5% (w/v) non-fat milk in 1x Dulbecco’s Phosphate Buffered Saline with Tween-20 (DPBST)) for 1 hour at room temperature. After three washes in DPBST, membranes were incubated in primary antibody (1:1000 Mouse Anti-FLAG primary antibodies (Sigma, Catalog #F1804), 1:80,000 Rabbit Anti-GroEL primary antibody (Sigma, Catalog #G6532)) overnight at 4ºC. After three washes in DPBST, membranes were then incubated in 1:20,000 secondary antibodies (Goat Anti-Mouse fluorescent secondary antibody 680RD (Licor, Catalog #926-68070), Goat Anti-Rabbit fluorescent secondary antibody 800CW (Licor, Catalog #926-32211) for 60 min at room temperature. After three washes with TBST, results were imaged on a LiCor at 700 nm and 800 nm emission wavelengths.

Isolation, scaling validation and sequencing of mutant phages that overcome defense

To isolate mutant phages that escaped *Kae*CanABC-mediated defense, plaque assays were performed as described above. After overnight incubation at 30ºC, single plaques from the highest phage dilution where plaques were visible were picked and resuspended in 50 µL of SM buffer. To confirm the ability of these “escaper” phages to avoid targeting by the *Kae*CanABC system, putative escaper phages and the ancestor phage (i.e., a plaque formed by T4 on a lawn of cells encoding the empty vector) were further diluted in tenfold serial dilutions in SM buffer, and re-plaqued on lawns of *E. coli* encoding either an empty vector or the *Kae*CanABC defense operon. After overnight incubation at 30ºC, plaque forming units (PFUs) were counted and used to estimate the fold-defense (ratio of PFUs on empty vector to PFUs on *Kae*CanABC) for each phage.

Escaper phages that plaqued similarly on cells expressing *Kae*CanABC and cells carrying the empty vector were further propagated to obtain high titre phage stocks for sequencing as described above. Phage genomic DNA was extracted from 200 µL of concentrated phage lysate using the Norgen Biotek Phage DNA Isolation Kit (# 46800), following the manufacturer’s instructions with the following minor modifications. Briefly, bacterial DNA was removed from the phage lysate by incubating it with 20µL of RQ1 DNAse I (PROMEGA M610A, 1u/µL) at room temperature for 15 minutes, followed by a 5-minute incubation at 75ºC to inactivate the enzyme. Then, the phages were lysed by treatment with 500 µL of Norgen Lysis Buffer supplemented with 10 µL of Proteinase K (New England Biolabs P8107S, 800u/mL), and subsequent incubation at 55ºC for at least 30 minutes, followed by inactivation of the enzyme by incubation at 65ºC for 15 minutes. After addition of 320 µL of isopropanol, the lysate was bound to the Norgen column and gDNA purification followed as recommended by the manufacturer. gDNA was double-eluted from the column in a total of 300 µL of ddH_2_O.

gDNA short read sequencing was performed by SeqCoast Genomics (short reads; 200 Mbp/1.3 million reads per sample). The ancestor phage reference genome was assembled by aligning reads to the phage T4 reference genome (GenBank: AF158101.6) to obtain a consensus sequence. Escaper phage genomes were assembled using this ancestor phage reference genome, and then re-mapped to it, allowing for the identification of mutations unique to escaper phages.


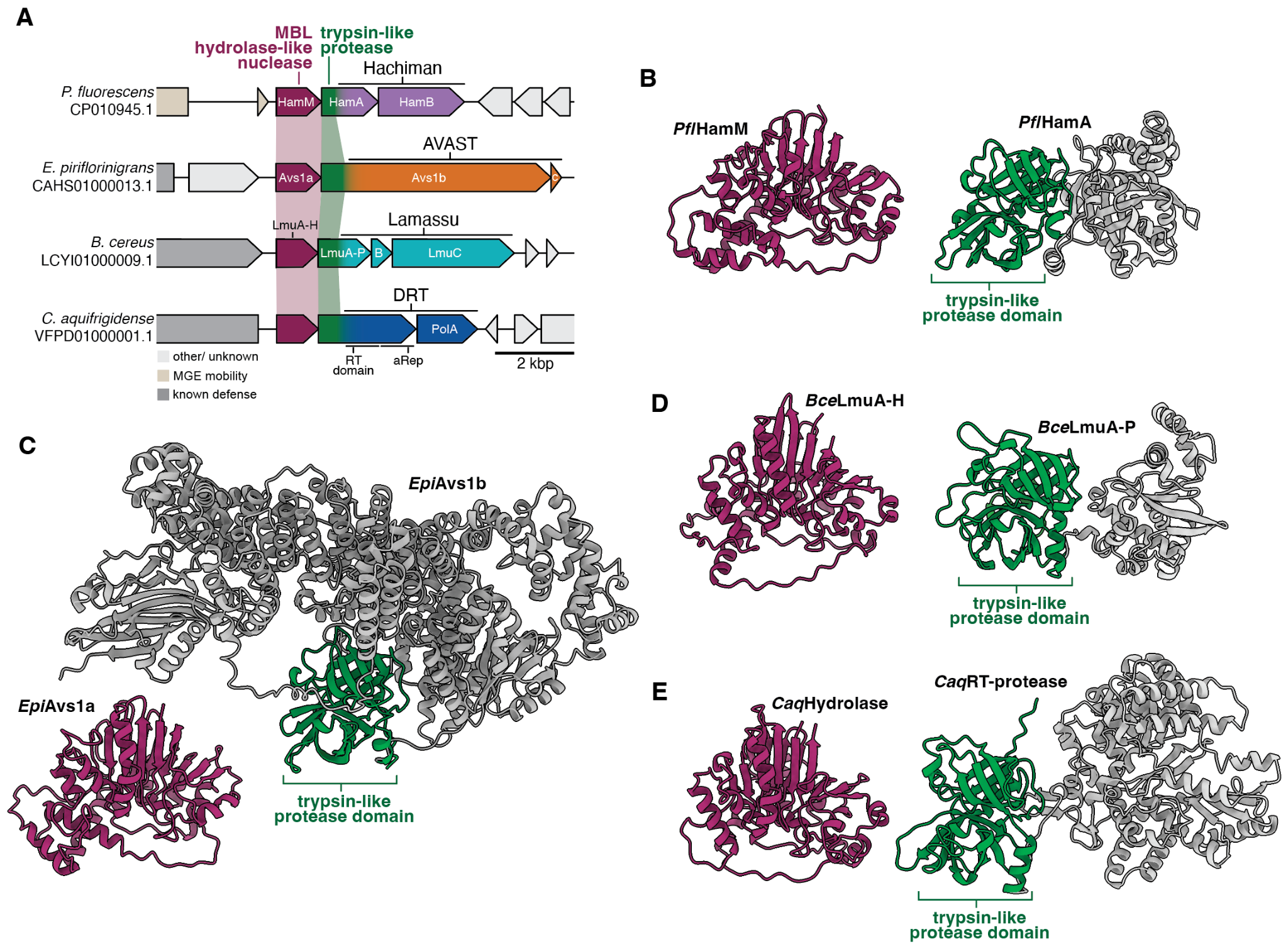


**Fig. S1. Structural comparison of nuclease-protease pairs in known defense systems.** (**A**) Loci illustrating the genetic architecture of multiple defense systems encoding an N-terminal MBL hydrolase-like nuclease and partnering trypsin-like protease, as in Fig. 1A. Annotations for gene context and gene names are shown. (**B**-**E**) AlphaFold3 predicted protein structures for nuclease and protease-fusion genes in Hachiman, AVAST, Lamassu, and DRT (UG9) systems. In each case, protein structures were predicted using loci from (A). Predicted structural snapshots are aligned to show homology.


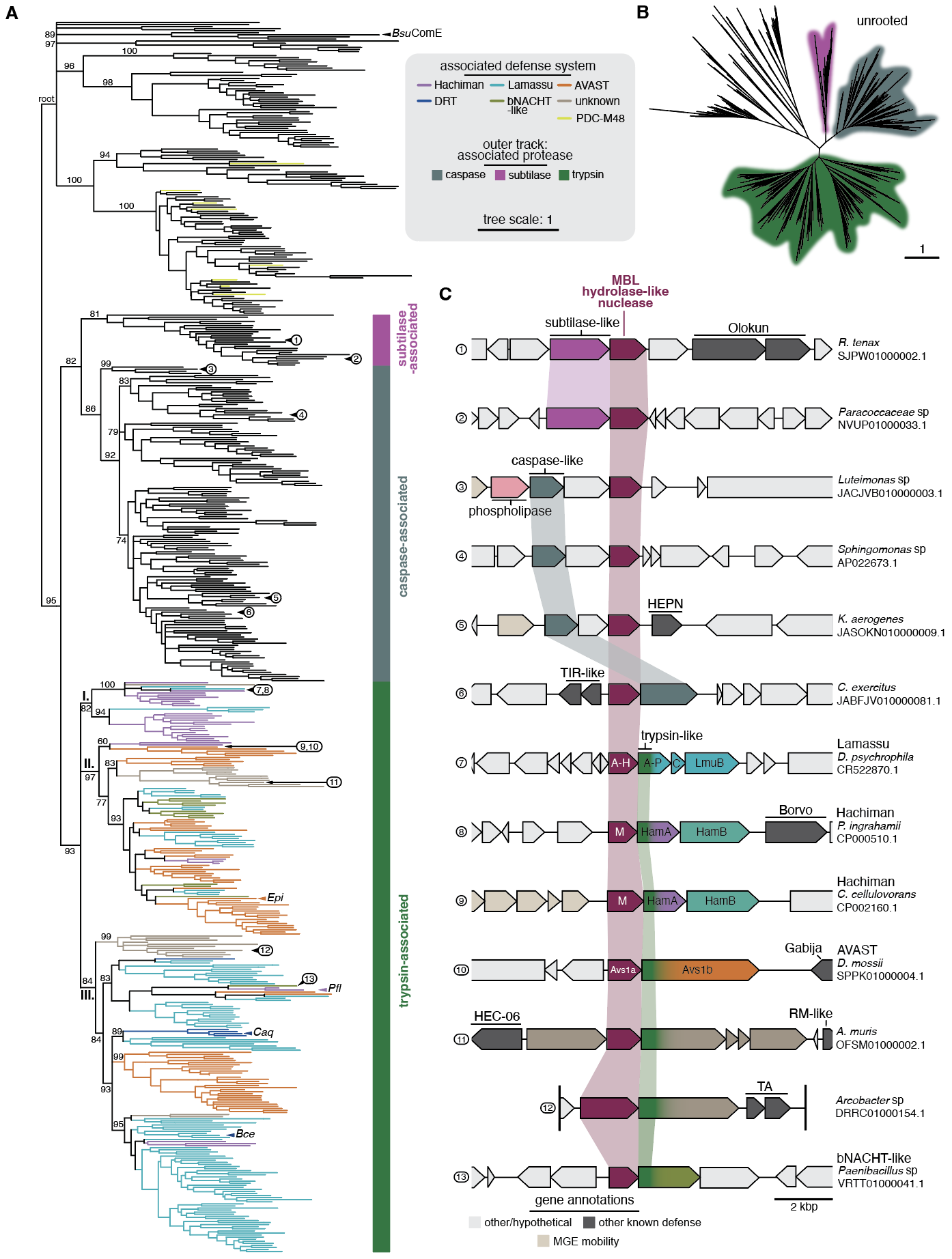


**Fig. S2. Modular evolution of defense-associated MBL hydrolase-like nucleases.** (**A**) Phylogenetic tree of diverse MBL hydrolase-like nuclease sequences in bacteria. The phylogenetic tree is rooted at a large clade containing MBL hydrolase-like nucleases without known protease associations which encode ComE nuclease domains. Tree leaves are colored according to inclusion of the predicted nuclease in known defense systems as determined by PADLOC search of a 20 kbp genomic context. The outer track indicates association with nearby protease families. Three major operonic protease families - subtilase-, caspase-/CHAT- and trypsin-family proteases were detected. Branches with support values < 60 are deleted, resulting in polytomy. Select bootstrap support values are shown. The clade containing trypsin-associated nucleases is shown as a subtree in Fig. 1B. A subtree of protease-associated nucleases is shown with clades collapsed in Fig. 4A. (**B**) Unrooted form of phylogenetic tree from (A). Colored ranges indicate associated proteases as in (A). (**C**) Genomic loci of nucleases from the phylogeny in (A). Select loci from each major protease-associated clade are labelled in A. Core defense genes, proteases, other detected defense systems in the gene neighborhood and genes associated with mobile genetic element mobility are annotated as in (A) and according to the key below.


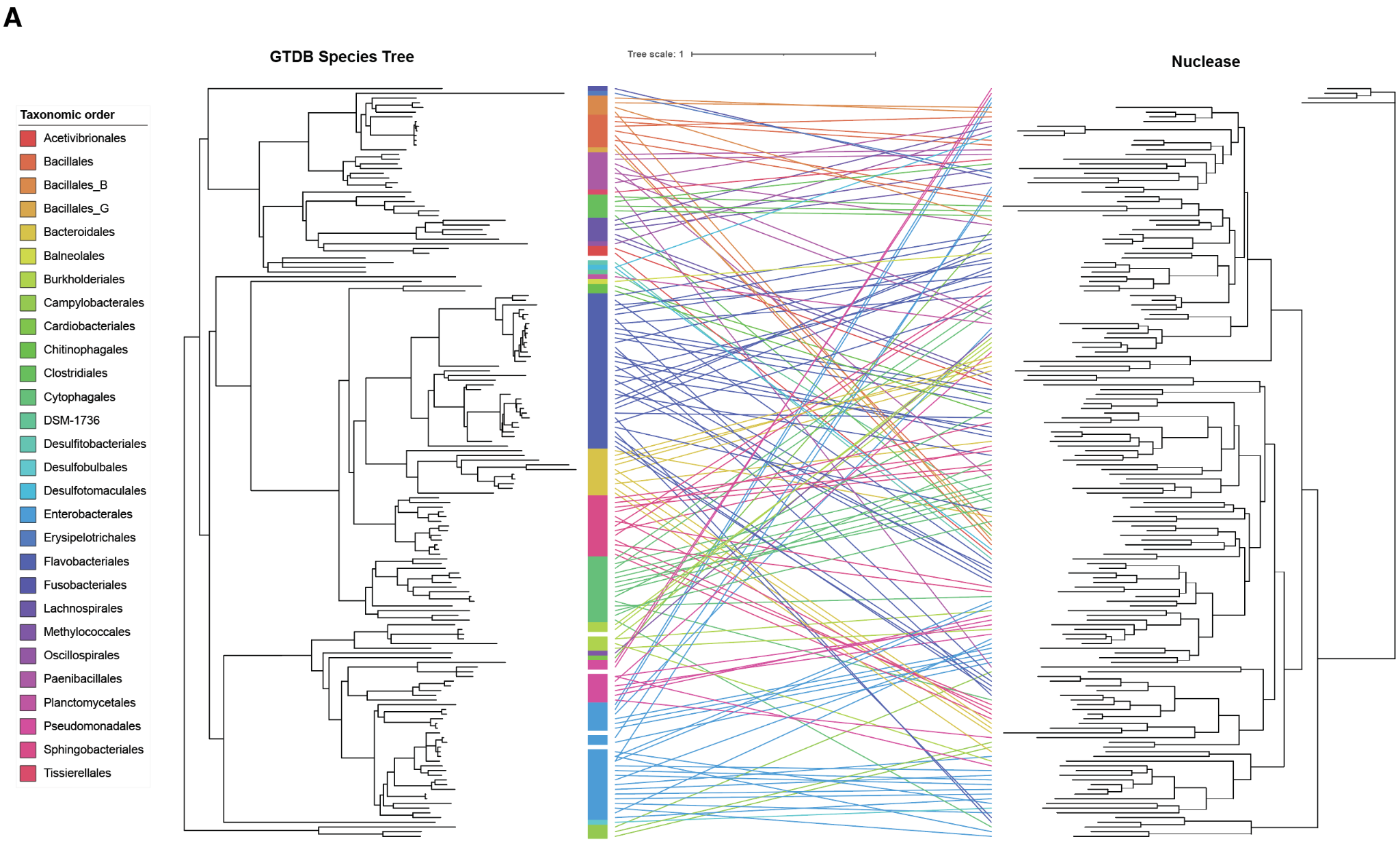


**Fig. S3. Comparison between species and nuclease trees and reconciliation analysis.** (**A**) Tanglegram comparing a species tree extracted from the Genome Taxonomy Database (GTDB) and select homologs of MBL hydrolase-type nucleases encoded by these species (see Methods). Taxonomic order is indicated by the species tree outer track. Lines connecting leaves show which species encode the corresponding nuclease and serve as a visualization for topological incongruence. Formal reconciliation analyses (GeneRax, UndatedDTL, see Methods) suggest that horizontal transfer and gene loss, but not duplication, primarily account for the evolutionary history of these genes (duplication = 1e-07, loss = 0.266004, transfer = 0.413985).

**
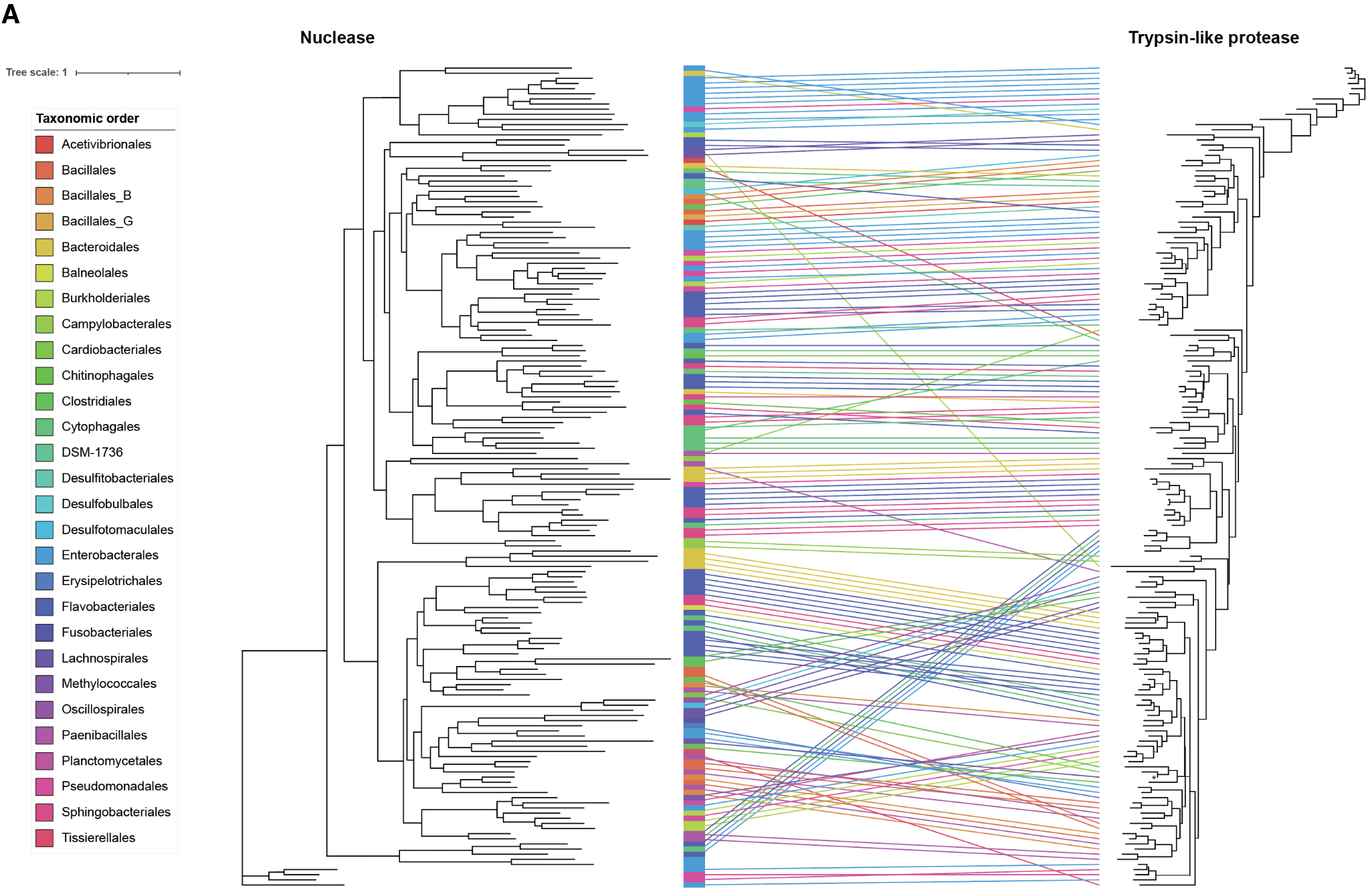
**

**Fig. S4. Comparison of nuclease and trypsin-like protease phylogenies.** (**A**) Tanglegram comparing phylogeny of select homologs of MBL hydrolase-type nucleases and a phylogeny of the corresponding trypsin-like protease (see Methods). Taxonomic order is indicated by the nuclease tree outer track. Lines are colored according to nuclease taxonomy and connect to co-encoded proteases.


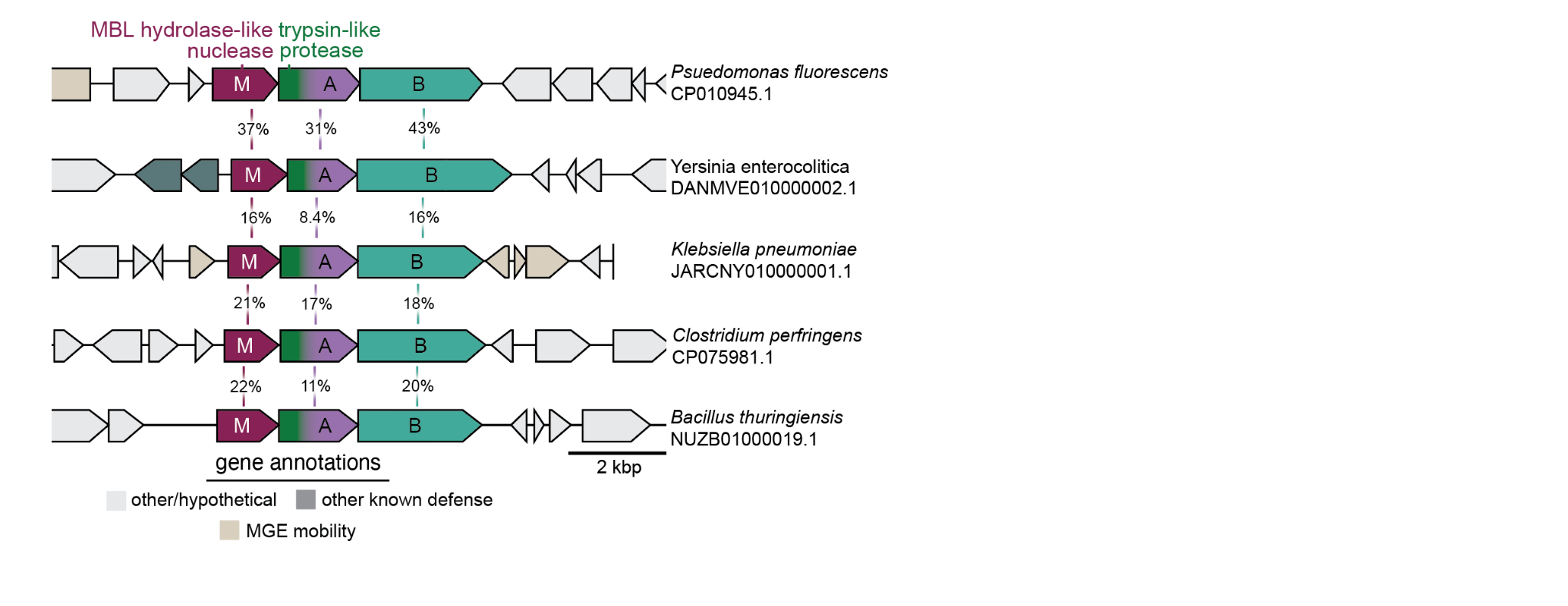


**Fig. S5. Nuclease-protease Hachiman systems are highly diverse.** Genomic loci encoding nuclease-protease Hachiman systems from different clades of the nuclease phylogeny in fig. S2A. Percent identity between different genes are shown between loci. *P. fluorescens* and *Y. enterocolitica* sequences originate from the same clade.


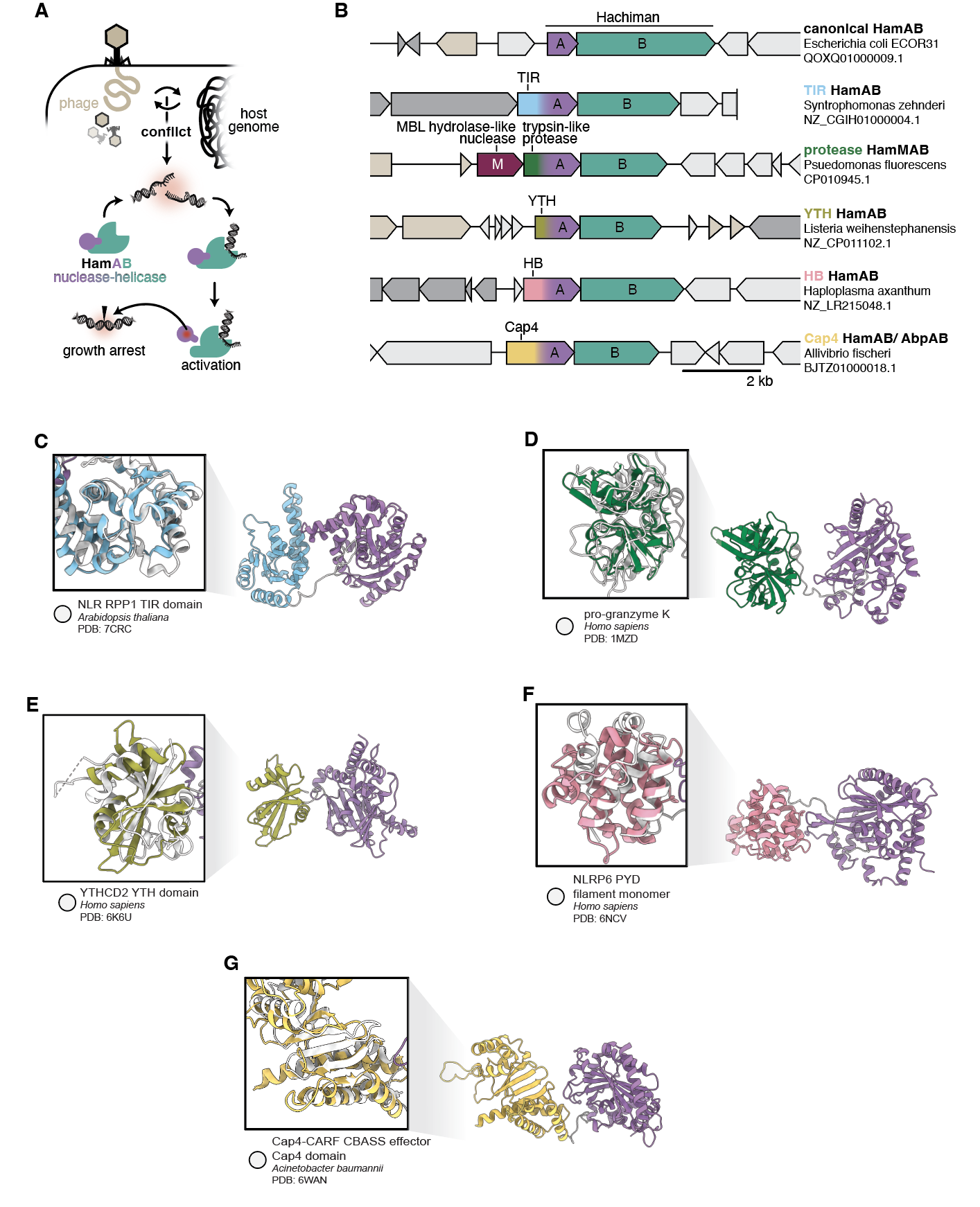


**Fig. S6. Hachiman HamA acquired multiple additional domains.** (**A**) Model for Hachiman antiphage defense. (**B**) Loci illustrating the genetic architecture of canonical and fusion variant Hachiman systems. The five fusion-HamA systems identified are: Toll/interleukin-1 receptor (TIR) domain-HamA, trypsin protease-HamA with upstream metallo‐β‐lactamase fold hydrolase-like nuclease (HamM), YT521-B homology (YTH) domain-HamA, helical bundle (HB) domain-HamA, and CBASS activated protein 4 (Cap4) domain-HamA or AbpA. (**C**-**G**) AlphaFold structural predictions of domain-HamA fusions shown in (B). Inset boxes show structural superpositions with DALI-predicted homologous experimental structures, which are shown in white and are indicated below in each case.


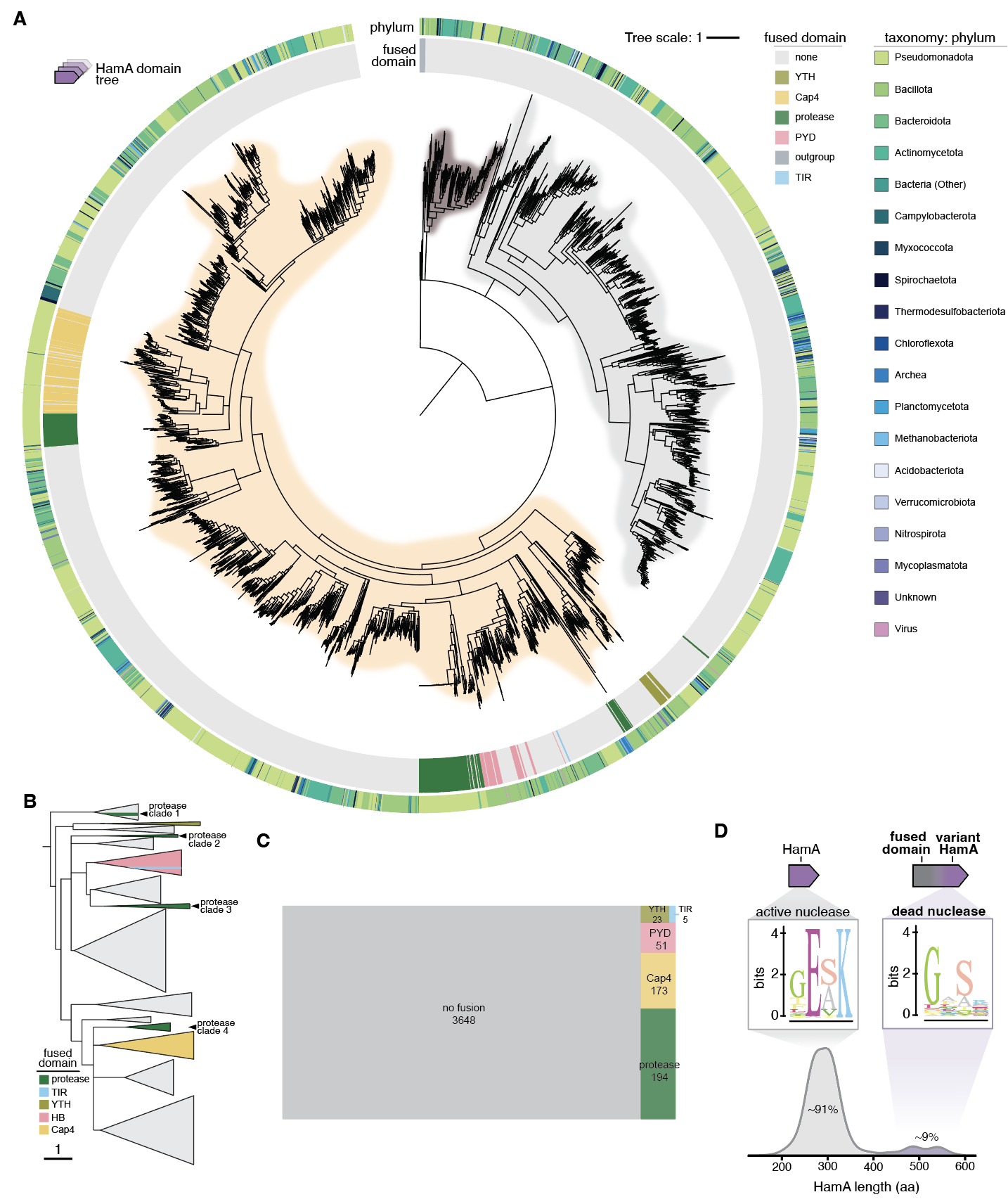


**Fig. S7. HamA domain phylogeny and loss of nuclease activity.** (**A**) Phylogenetic tree of 4,094 HamA domain sequences. The tree is rooted at a clade containing 10 homologs of the *P. aquatile* type IIS restriction endonuclease domain. The inner track indicates presence of a fusion domain, but these were excluded from phylogenetic analysis to ascertain the phylogeny exclusively of the HamA domain. The outer track indicates associated phyla. Branches with support values < 60 are deleted, resulting in polytomy. (**B**) Cartoon of the fusion containing clade in (A), with clades collapsed and color-coded to indicate fusion content. (**C**) Fraction of HamA sequences encoding fusions. (**D**) Sequence logos of the nuclease active site for canonical versus domain fusion HamA variants are shown above a density plot showing the distribution of HamA protein lengths across HamA diversity. HamA variants were manually curated from all HamA domain sequences from panel (A) and aligned.

**
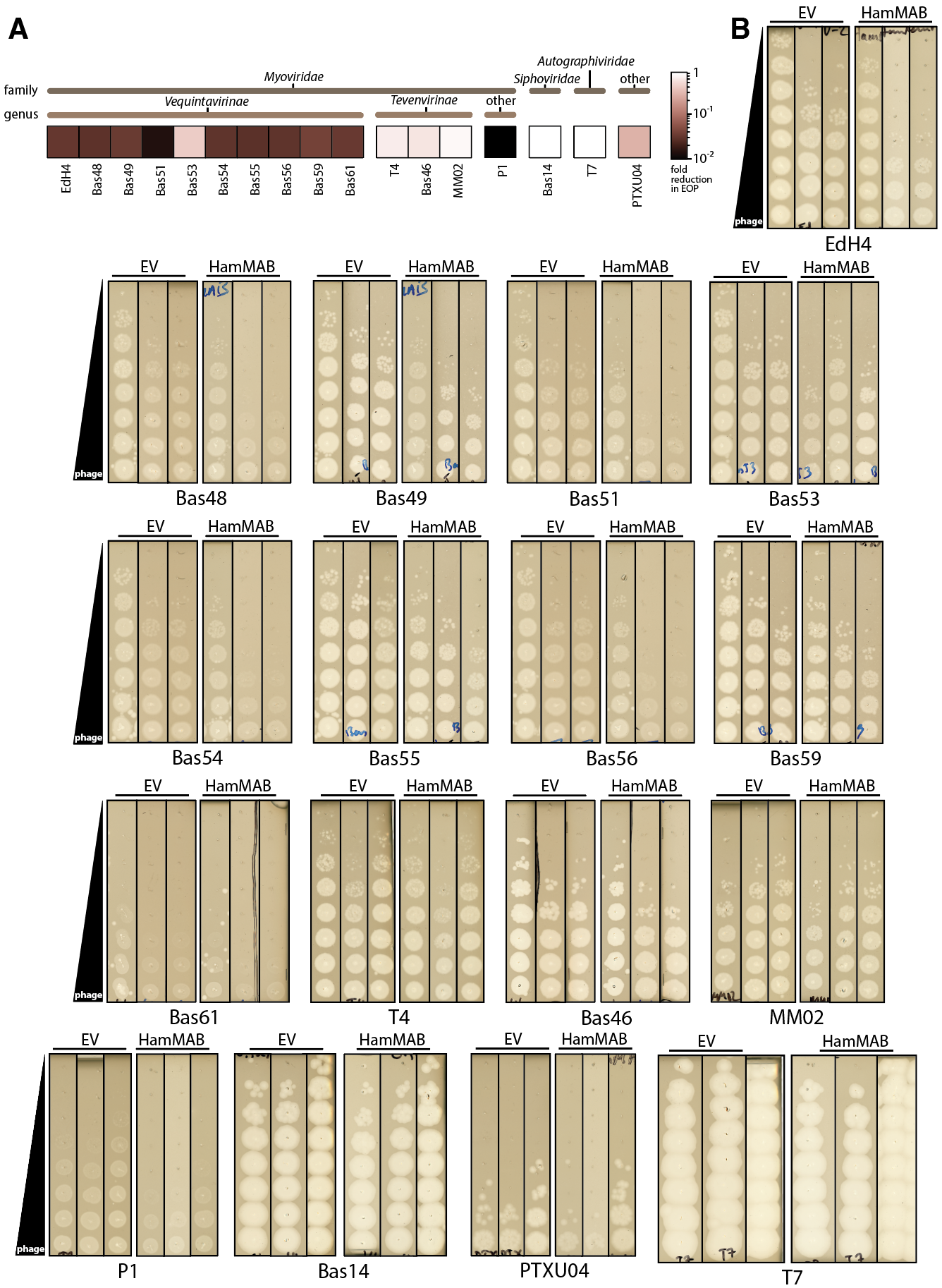
**

**Fig. S8. *Pseudomonas fluorescens* HamMAB is antiphage.** (**A**) Heatmap of the efficiency of plating of diverse phages on *E. coli* expressing *P. fluorescens* HamMAB. (**B**) Images of triplicate plaque assays where *E. coli* expressing *Pfl*HamMAB was challenged with diverse bacteriophages indicated. Experiments are summarized in (A).


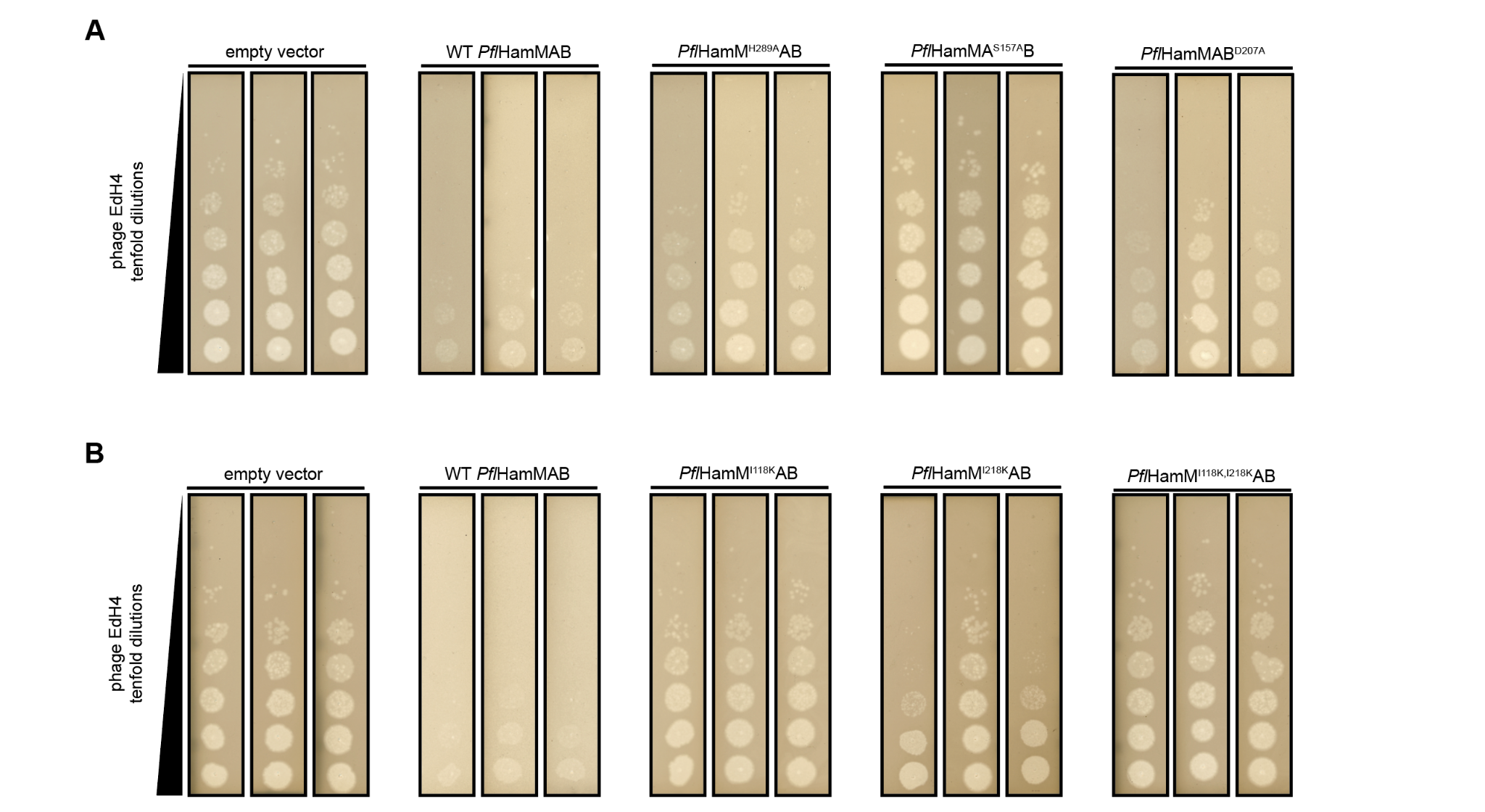


**Fig. S9. Plaque assays of *P. fluorescens* HamMAB mutants.** (**A**) Images of triplicate plaque assays where *E. coli* expressing *Pfl*HamMAB and catalytic mutants were challenged with tenfold dilutions of phage EdH4. (**B**) Images of triplicate plaque assays where *E. coli* expressing *Pfl*HamMAB and cut site mutants of HamM were challenged with tenfold dilutions of phage EdH4.


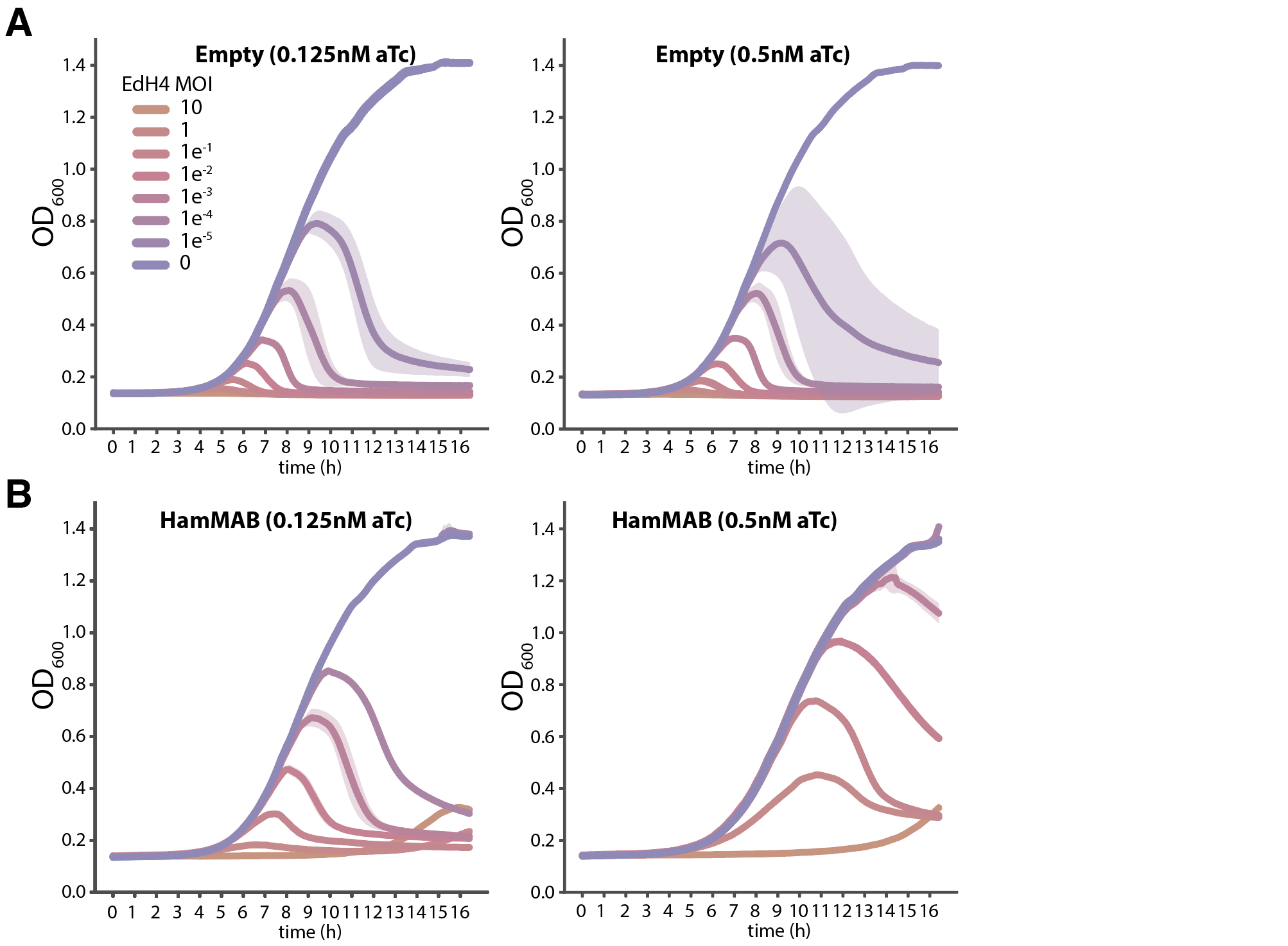


**Fig. S10. Complete liquid infection data for *Pfl*HamMAB.** (**A**) Growth curves of *E. coli* empty vector control during EdH4 infection at the specified multiplicity of infection (MOI) and level of induction. (**B**) Growth curves of *E. coli* expressing *Pfl*HamMAB during EdH4 infection at the specified multiplicity of infection (MOI) and level of induction. In all cases, data are shown as mean ± standard deviation (shaded area) across three independent biological replicates.


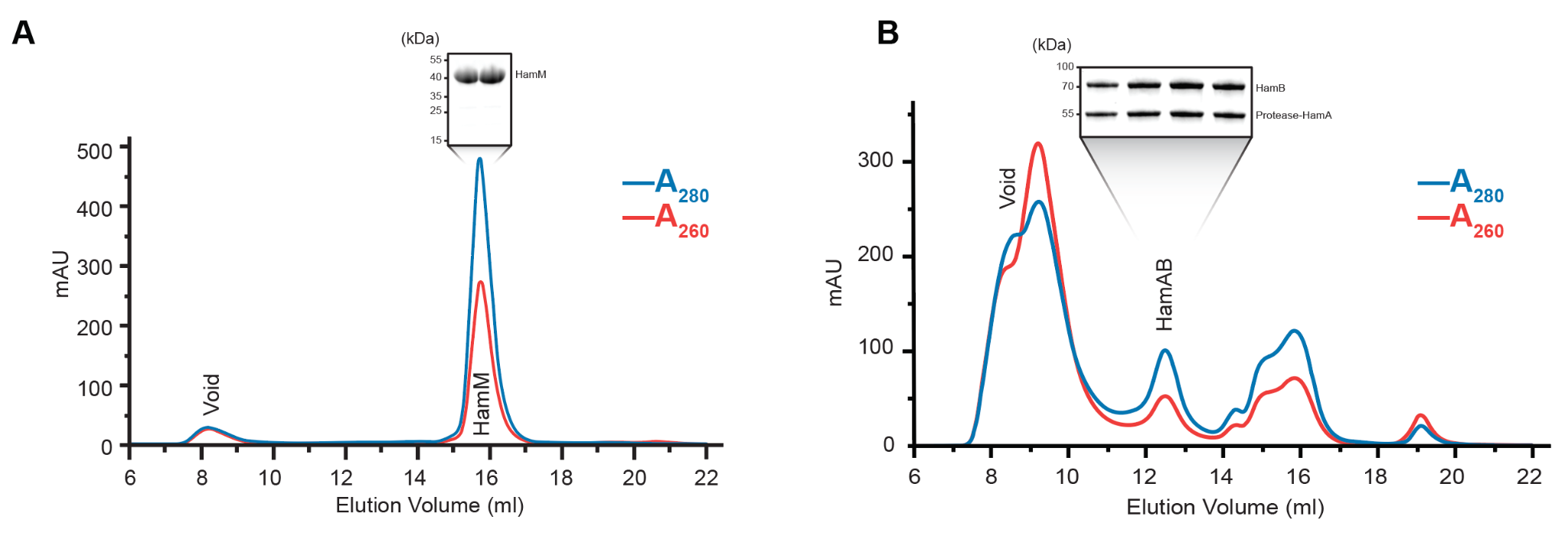


**Fig. S11. Purification of HamM and HamAB. (A)** Size exclusion chromatography trace of HamM after TEV protease treatment and removal of MBP by reverse immobilized metal affinity purification (IMAC). Corresponding peaks of elution fractions are run on a Coomassie PAGE gel. **(B)** Size exclusion chromatography trace of HamM after TEV protease treatment and removal of MBP by reverse IMAC. Corresponding peaks of elution fractions were run on a Coomassie PAGE gel.

**
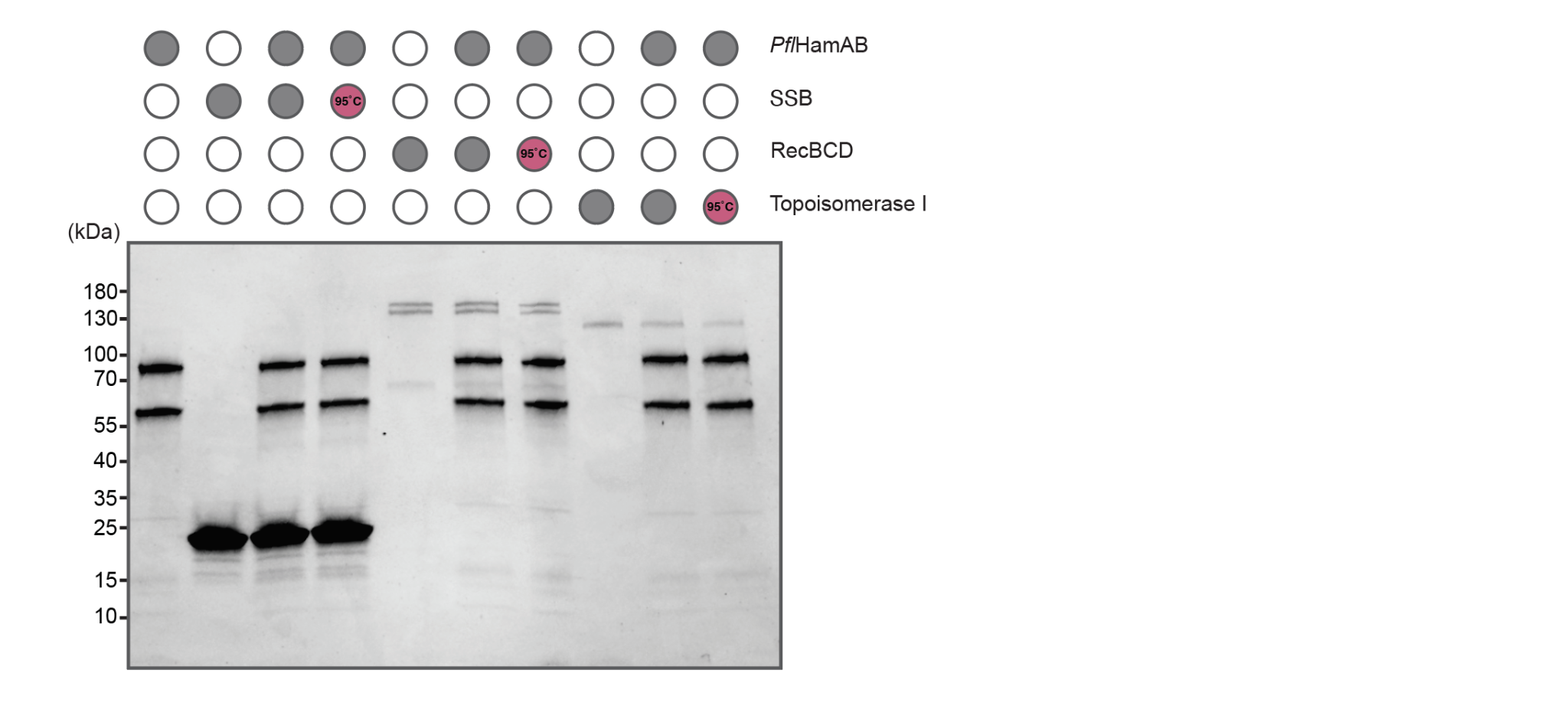
**

**Fig. S12. *In vitro* incubation of *Pfl*HamAB with Single-Stranded DNA Binding Protein (SSB), RecBCD, and Topoisomerase I.** *In vitro* reactions of HamAB with SSB, RecBCD, and Topoisomerase I visualized on a Coomassie PAGE gel. In lanes 4, 7, and 10 (from left to right), protein substrates were pre-boiled before addition of HamAB.

**
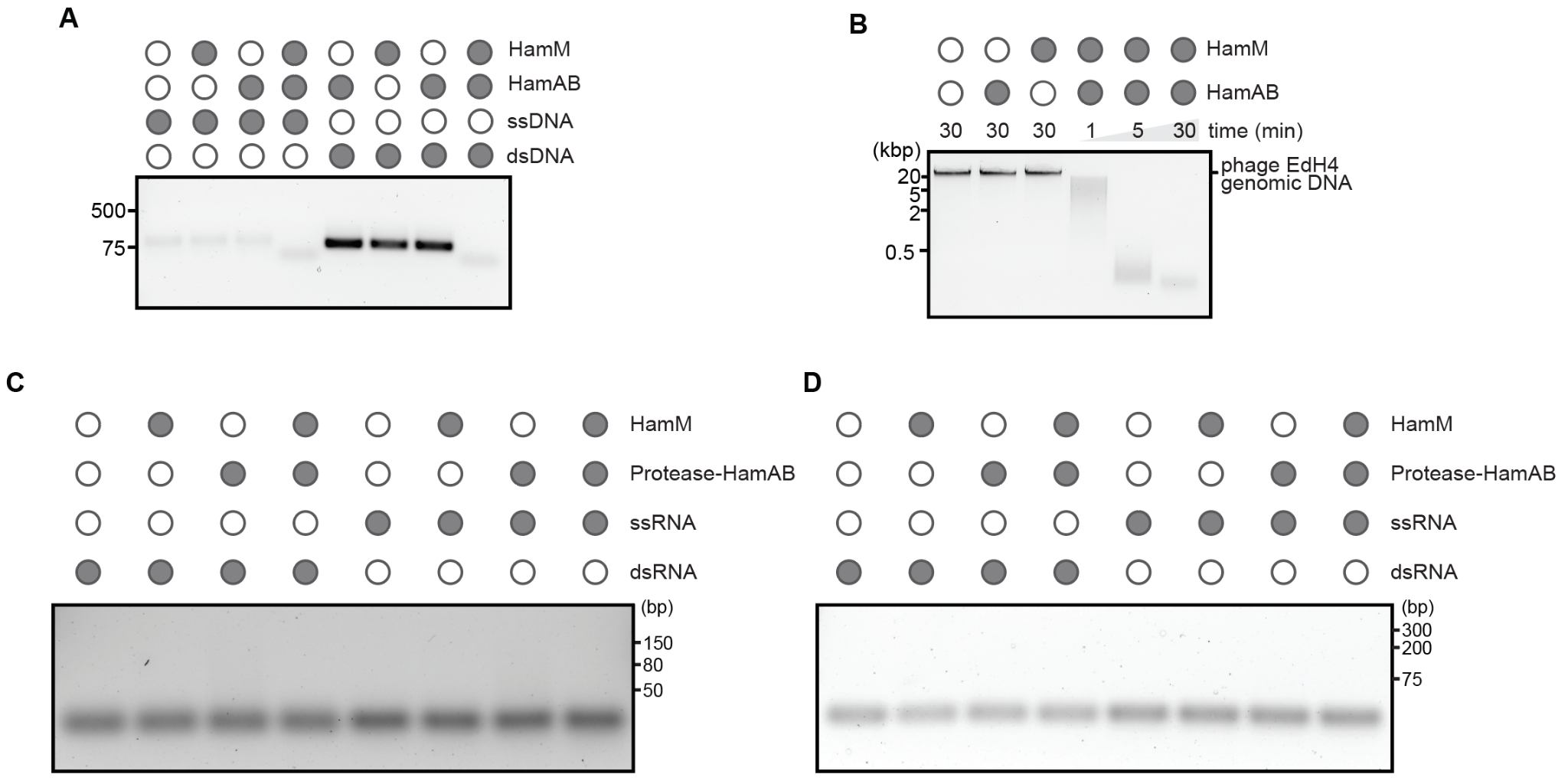
**

**Fig. S13. *In vitro* cleavage of various nucleic acids by *Pfl*HamMAB. (A)** *In vitro* reactions of HamM and HamAB against ssDNA and ssDNA, visualized on a 2% agarose gel stained with SYBR Gold. **(B)** *In vitro* phage EdH4 gDNA cleavage assay with HamM and HamAB with time course. **(C)** *In vitro* reactions of HamM and HamAB against dsRNA and ssRNA, visualized on a 2% agarose gel stained with SYBR Gold. **(D)** *In vitro* reactions of HamM and HamAB against dsRNA and ssRNA in buffer supplemented with 1mM ZnCl_2_, visualized on a 2% agarose gel stained with SYBR Gold. For a summary of oligonucleotide substrates, see Table S6.


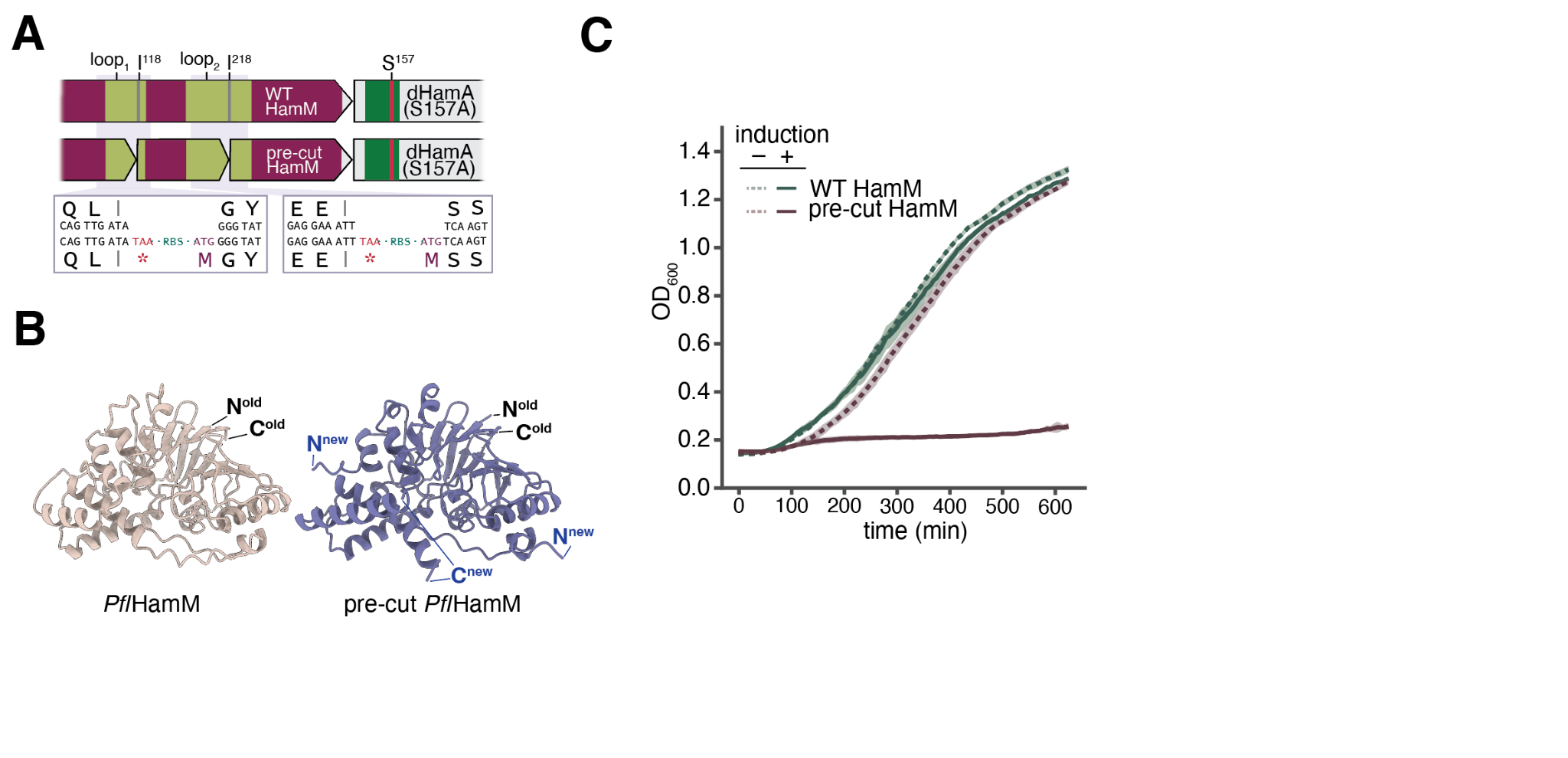
**Fig. S14. Pre-cut HamM is constitutively toxic to host cells.** (**A**) Diagram of comparing portions of the wildtype *Pfl*HamMAB locus with the ‘pre-cut’ version of the HamM in HamA protease-dead backgrounds. DNA and resulting protein sequences are shown as insets below loop 1 and loop 2 proteolysis insertion regions to illustrate how breaks in the peptide backbone were programmed. (**B**) AlphaFold3 predicted structures of wildtype *Pfl*HamMAB and ‘pre-cut’ tripeptide. The locations of the new N- and C-termini are shown. (**C**) Growth curves of *E. coli* expressing either native *Pfl*HamMAB or ‘pre-cut’ *Pfl*HamMAB, each with a deactivated protease domain (S157A), with or without induction. Data are shown as mean ± standard deviation (shaded area) across three independent biological replicates.

**
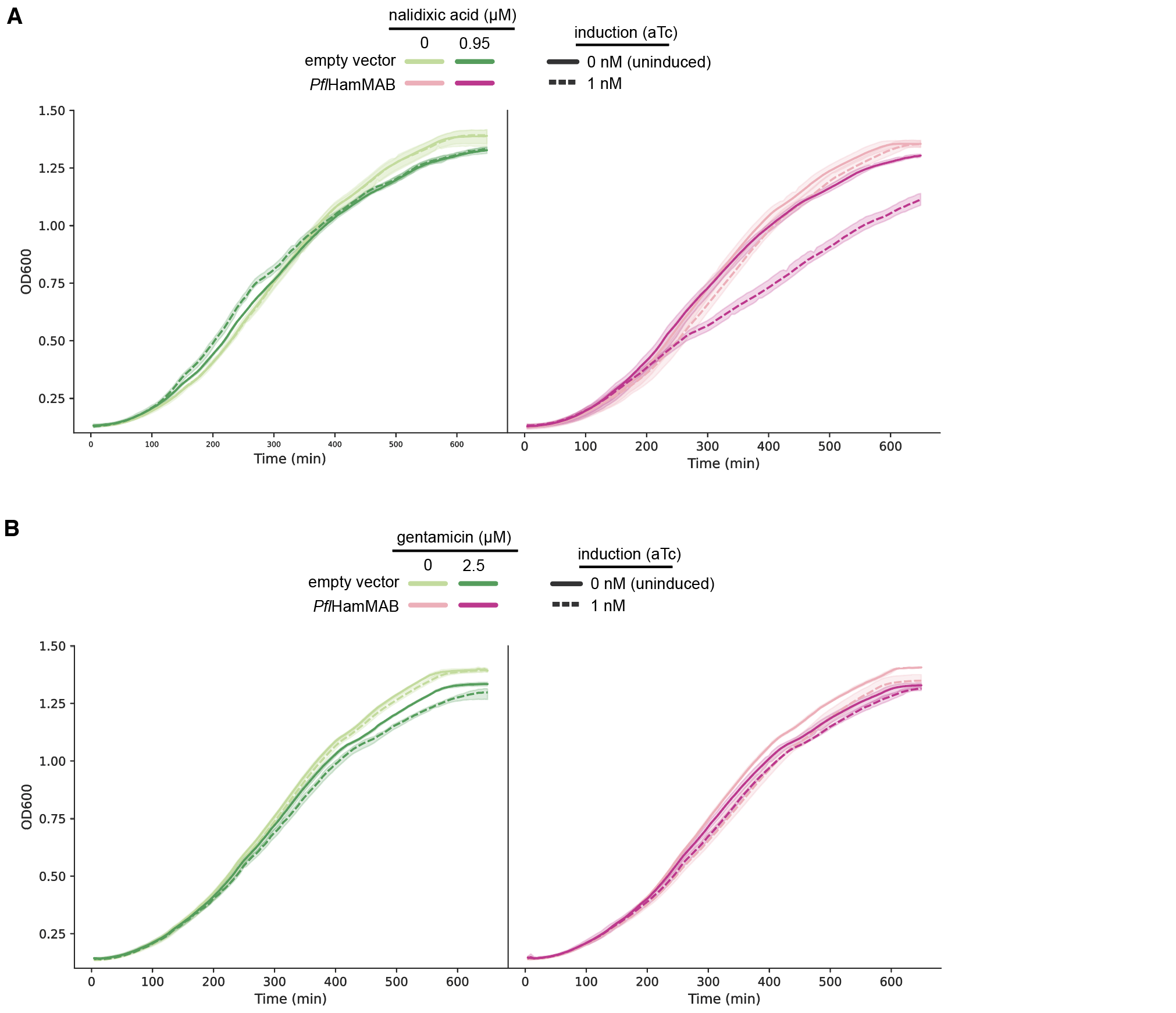
**

**Fig. S15. Drug-induced *Pfl*HamMAB toxicity.** (**A**) Growth curves of *E. coli* expressing either native *Pfl*HamMAB or an empty vector control, with or without the indicated concentrations of nalidixic acid and inducer (anhydrotetracycline). (**B**) Growth curves of *E. coli* expressing either native *Pfl*HamMAB or an empty vector control, With or without the indicated concentrations of gentamicin and inducer (anhydrotetracycline). In all cases, data are shown as mean ± standard deviation (shaded area) across three independent biological replicates.


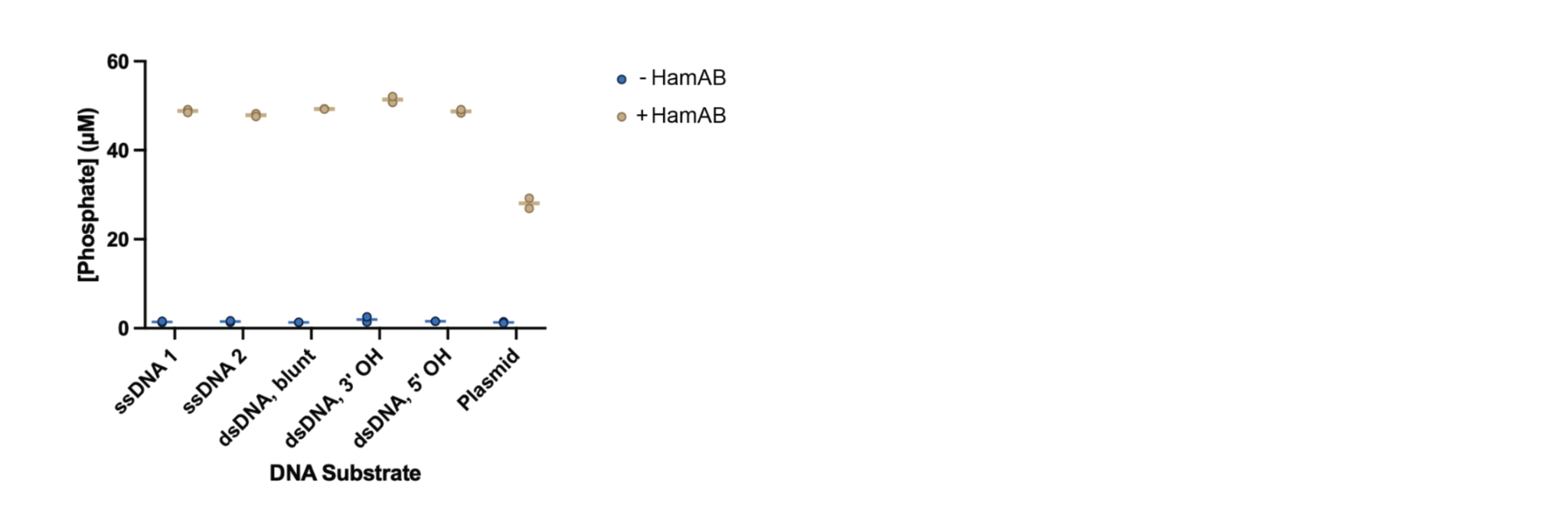


**Fig. S16. *In vitro* ATPase assay of *Pfl*HamAB.** Malachite green ATPase assays of HamAB with ATP against a panel of DNA substrates. Individual data points of two independent biological replicates are shown. For a summary of oligonucleotide substrates, see Table S6.


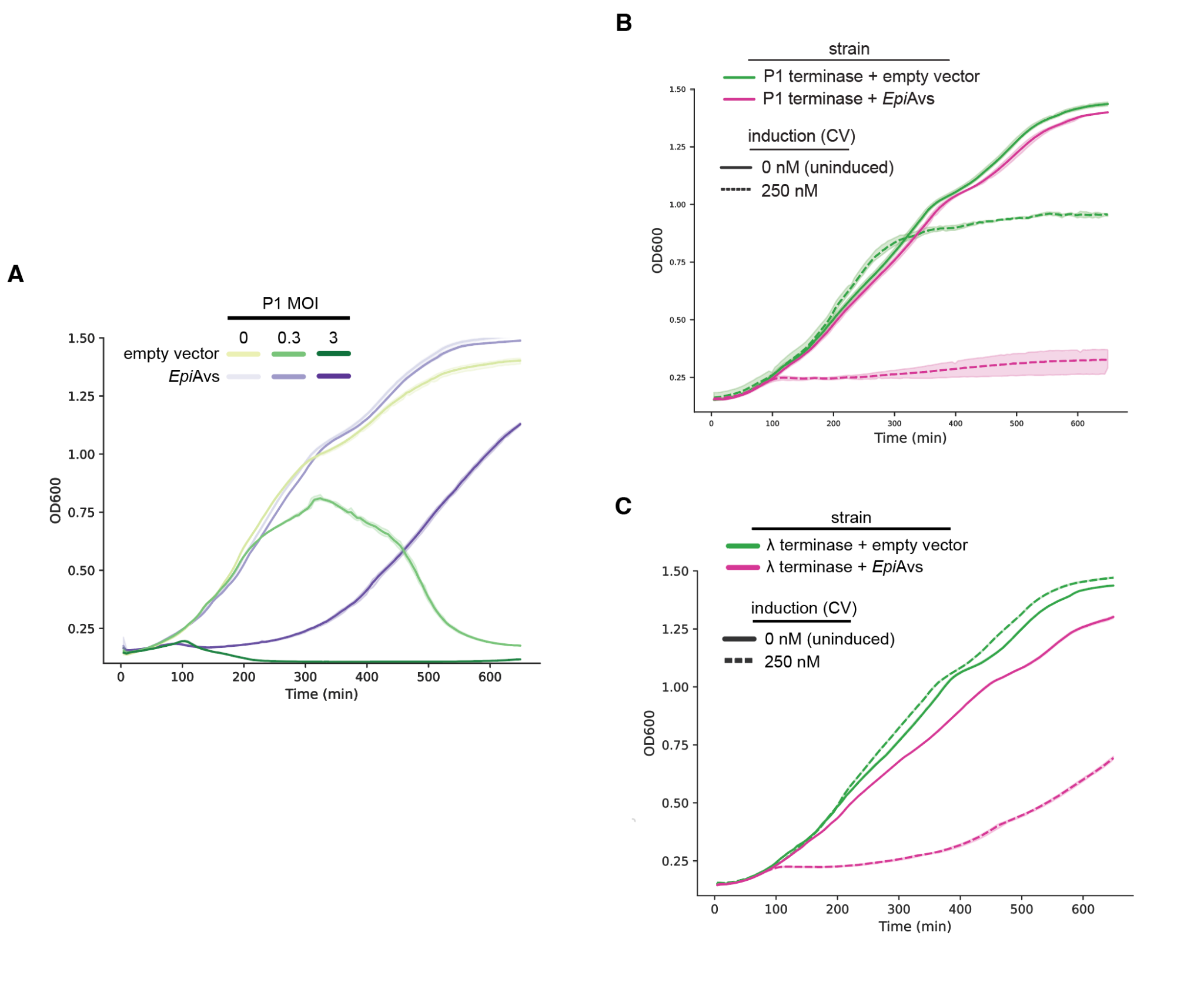


**Fig. S17. *Epi*Avs liquid infection and terminase-mediated activation.** (**A**) Growth curves of *E. coli* empty vector control or expressing *Epi*Avs during phage P1 infection at the specified multiplicity of infection (MOI). (**B**) Growth curves of *E. coli* coexpressing phage P1 terminase gene under control of the pJEX system with either *Epi*Avs or an empty vector control. Induction of the terminase gene with crystal violet (CV) is indicated with a dashed line. (**C**) Growth curves of *E. coli* coexpressing phage lambda terminase gene under control of the pJEX system with either *Epi*Avs or an empty vector control. Induction of the terminase gene with crystal violet (CV) is indicated with a dashed line. In every case, data are shown as mean ± standard deviation (shaded area) across three independent biological replicates.


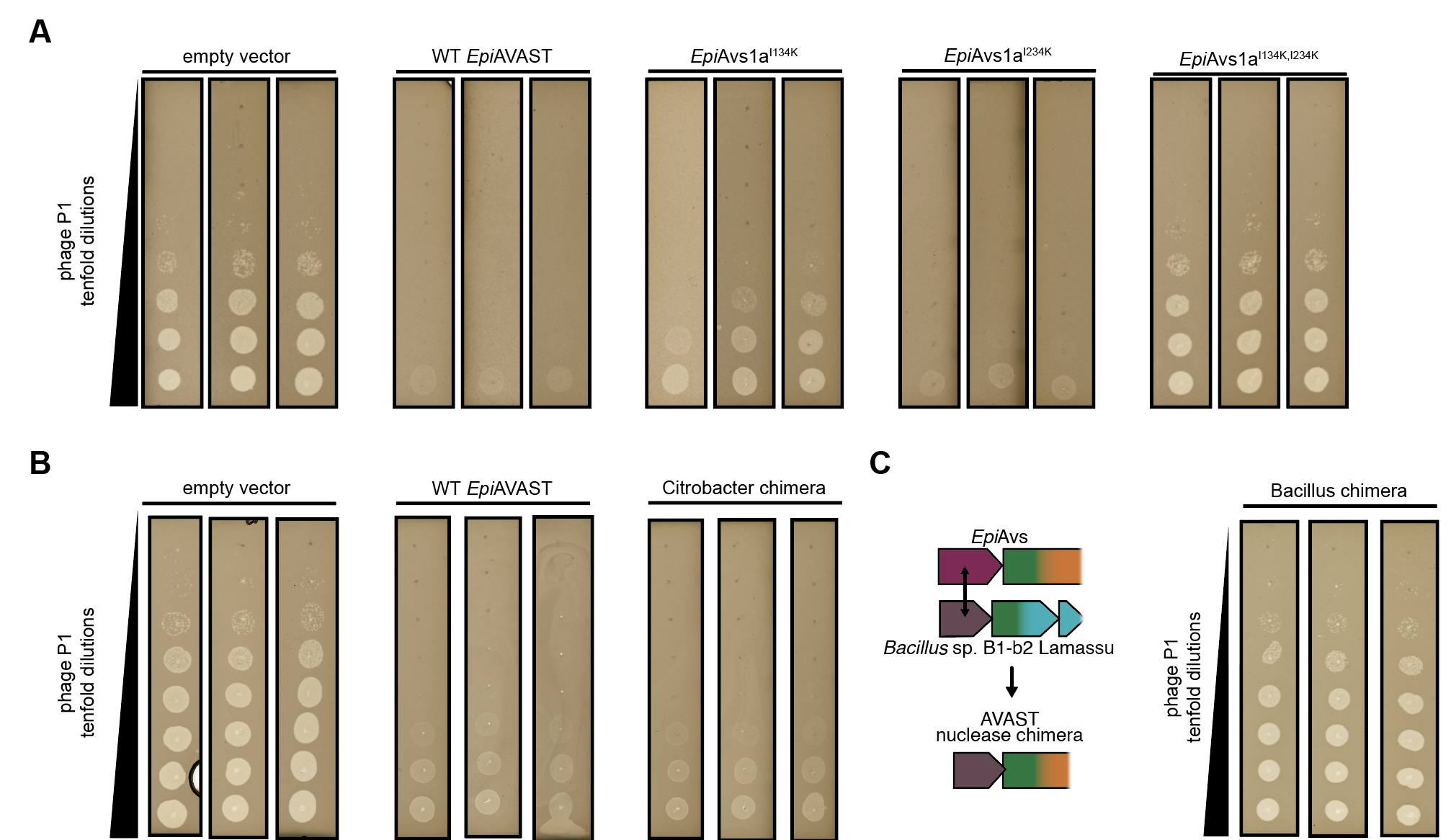


**Fig. S18. Plaque assays of *E. piriflorinigrans* AVAST and Avs1a mutants.** (**A**) Images of triplicate plaque assays where *E. coli* expressing *Epi*AVAST and catalytic mutants of Avs1a were challenged with tenfold dilutions of phage P1. (**B**) Images of triplicate plaque assays where *E. coli* expressing *Epi*AVAST and a chimeric *Epi*Avs system with an Avs1a nuclease from *Citrobacter* sp. ESBL3 (NZ_JAZEVZ010000002.1) were challenged with tenfold dilutions of phage P1. (**C**) Images of triplicate plaque assays where *E. coli* expressing a chimeric *Epi*Avs system with an LmuA-H nuclease homolog from *Bacillus* sp. B1-b2 (WEHU01000028.1) was challenged with tenfold dilutions of phage P1.


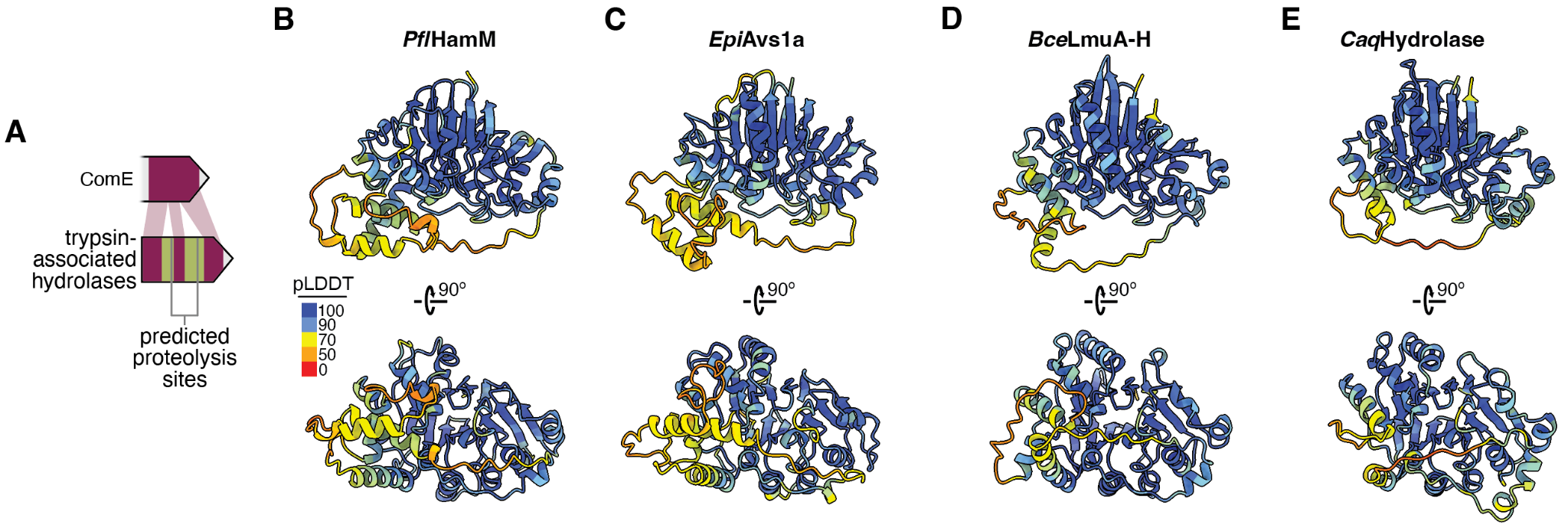


**Fig. S19. Structural comparisons of trypsin-associated nucleases.** (**A**) Model for the emergence of autoinhibitory ‘gating’ insertions (green) in trypsin-associated nucleases in antiphage systems. (**B**-**E**) AlphaFold3 predicted protein structures for nuclease genes in Hachiman, AVAST, Lamassu and DRT (UG9) systems, as in fig. S1 but with an additional view shown and colored by pLDDT. In each case, protein structures were predicted using loci from Fig. 1a. Predicted structural snapshots are aligned to show homology.


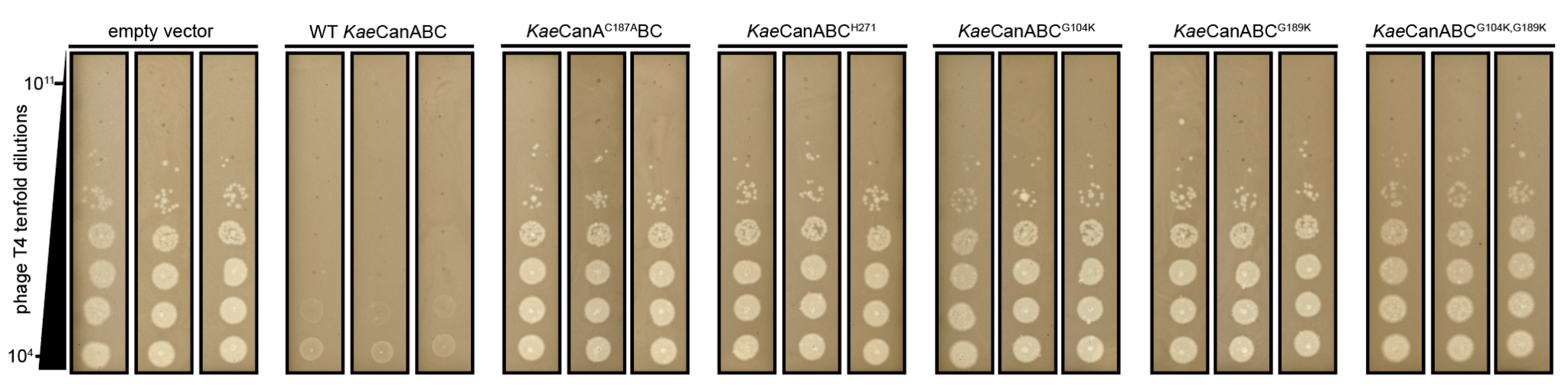


**Fig. S20. Plaque assays of *K. aerogenes* CanABC and mutants.** (**A**) Images of triplicate plaque assays where *E. coli* expressing *Kae*CanABC and various catalytic and cut site mutants were challenged with tenfold dilutions of phage T4.


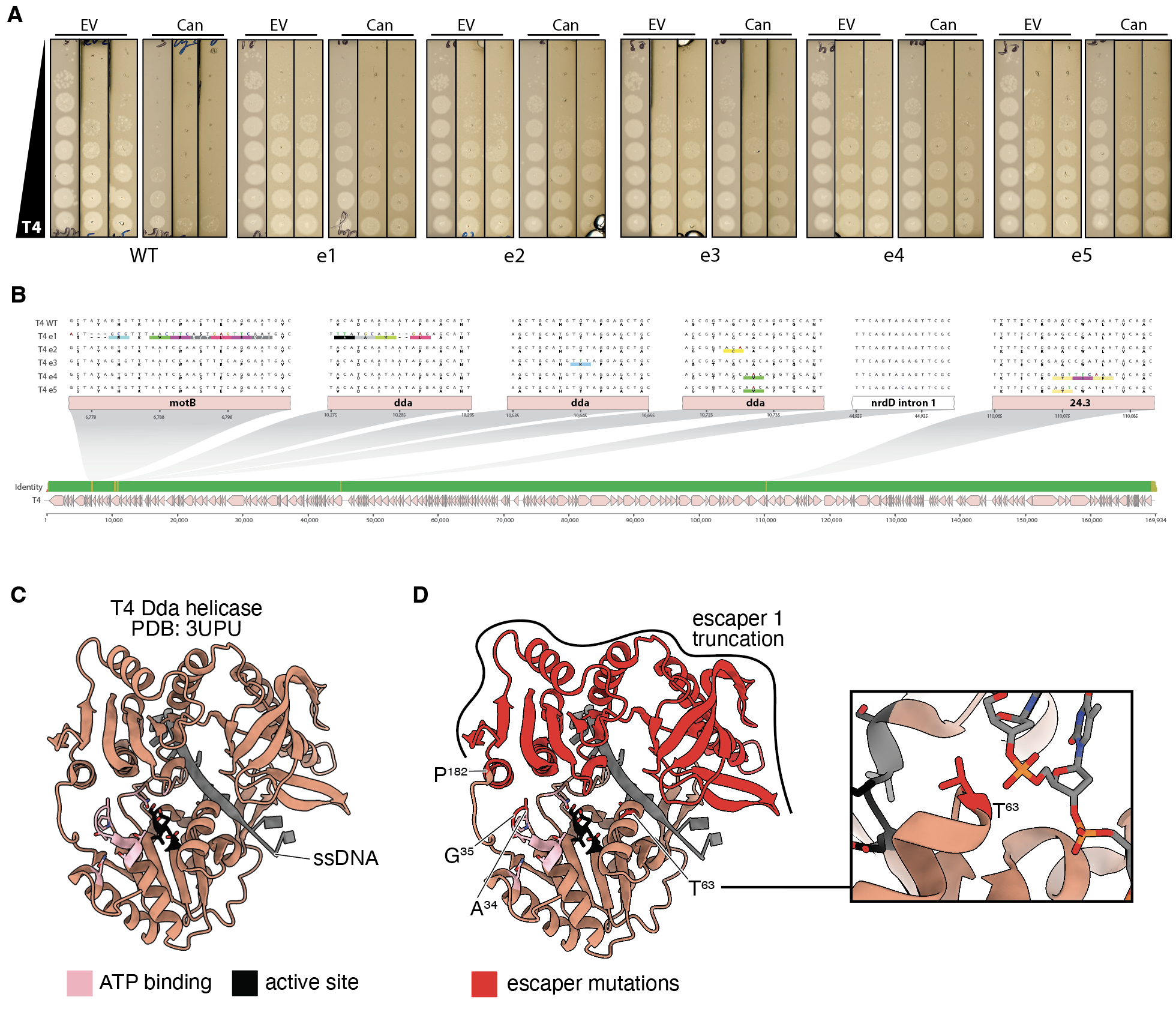


**Fig. S21. Phage escape of *Kae*CanABC involves mutation of the *dda* helicase.** (**A**) Images of triplicate plaque assays where *E. coli* expressing *K. aerogenes* CanABC was challenged with 10-fold dilutions of WT and isolated escaper phage T4. (**B**) Overview of the whole-genome alignments of WT and Canu-escaping T4 phages. (**C**) Overview of the T4 Dda helicase structure (PDB:3UPU) with ssDNA, with ATP binding and active site residues labeled. (**D**) Location of phage T4 escape mutants on the structure from (C). Locations of point mutations and truncations are shown in red, as in a close-up of residue T63 to demonstrate proximity to DNA.

**Table S1. (separate file)**

Cloned sequences used in this study.

**Table S2. (separate file)**

Summary of plasmids used in this study.

**Table S3. (separate file)**

Mass spetrometry results for HamM fragment 1 (high molecular weight).

**Table S4. (separate file)**

Mass spetrometry results for HamM fragment 2 (medium molecular weight).

**Table S5. (separate file)**

Mass spetrometry results for HamM fragment 3 (low molecular weight).

**Table S6. (separate file)**

Oligonucleotide constructs used in biochemical assays.
